# Supplementary material for: Terpenoid balance in Aspergillus nidulans unveiled by heterologous squalene synthase expression
Source: Sci Adv. 2024 Feb 21;10(8):eadk7416. doi: 10.1126/sciadv.adk7416 (PMC10881027; doi:10.1126/sciadv.adk7416)
Supplement: Supplementary file 1 — Figs. S1 to S27 Tables S1 to S4 References [file sciadv.adk7416_sm.pdf]

Science Advances
AAAS

Supplementary Materials for

Terpenoid balance in *Aspergillus nidulans* unveiled by heterologous squalene synthase expression

Sung Chul Park *et al.*

Corresponding author: Jin Woo Bok, jwbok@wisc.edu; Nancy P. Keller, npkeller@wisc.edu

Sci. Adv. **10**, eadk7416 (2024)
DOI: 10.1126/sciadv.adk7416

This PDF file includes:

Figs. S1 to S27
Tables S1 to S4
References

Supplementary Figures

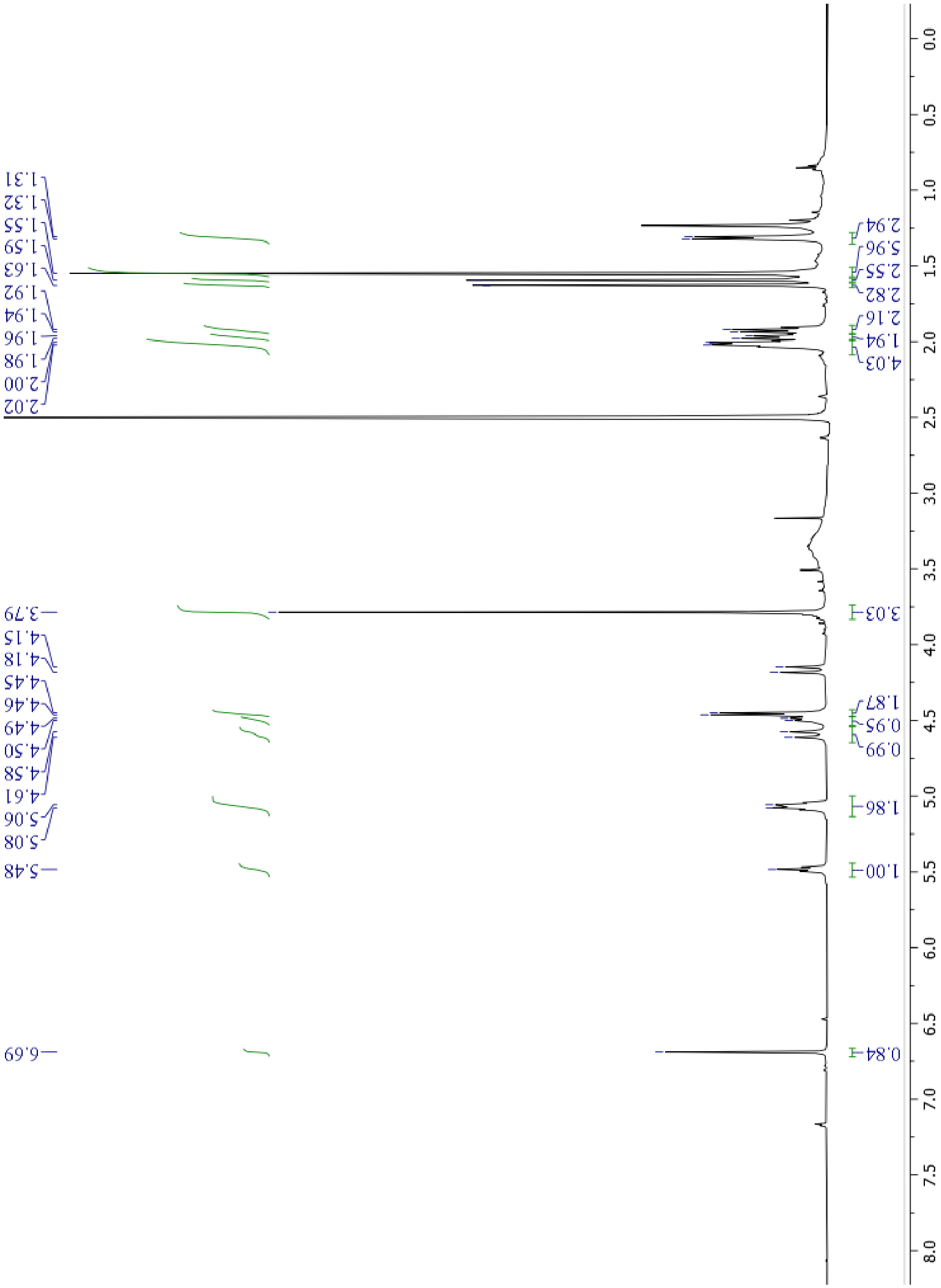

Fig. S1. The ^{1}H NMR (500 MHz, DMSO-d_6) spectrum of 1.

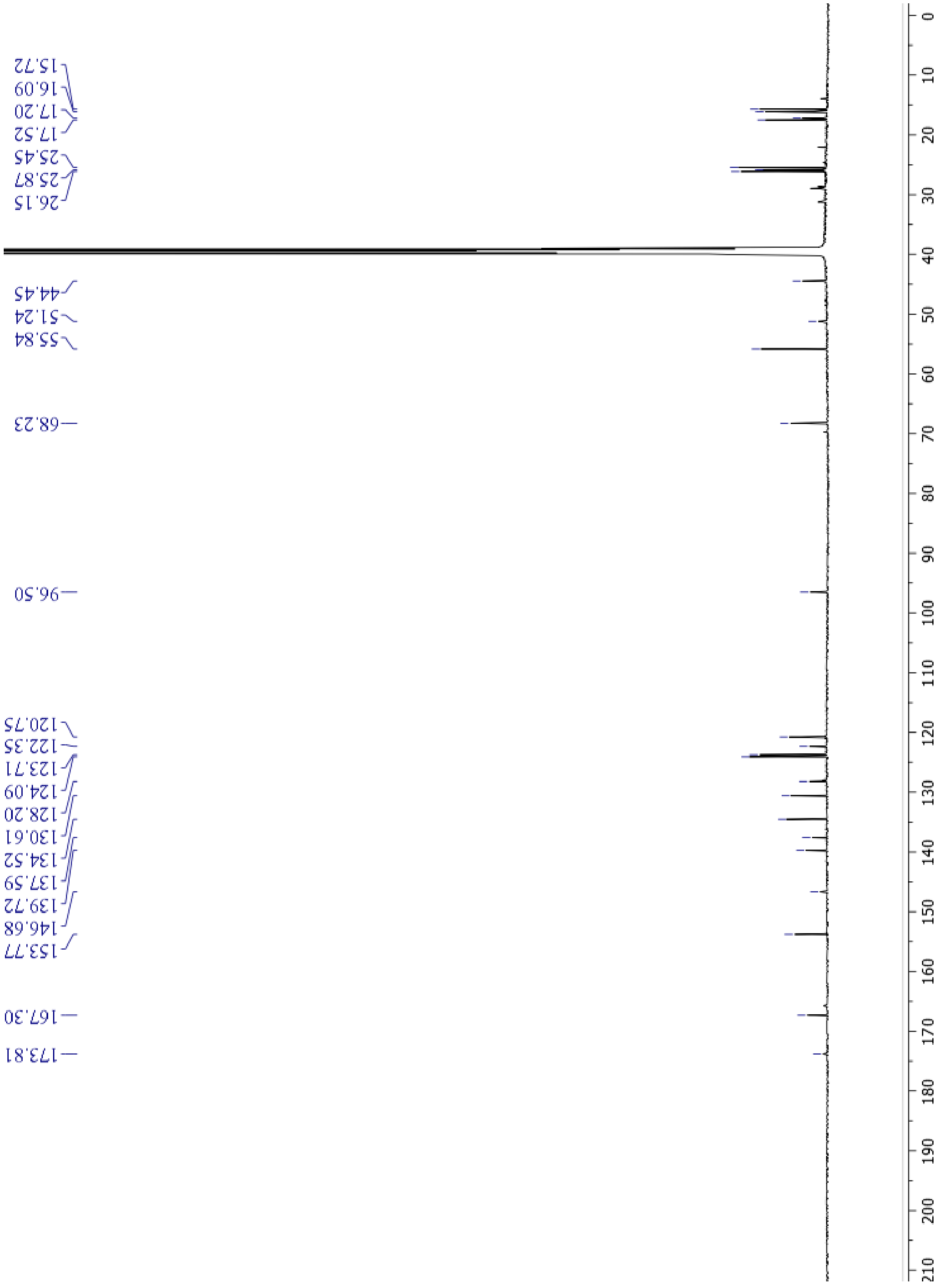

Fig. S2. The ^{13}C NMR (125 MHz, DMSO-d_6) spectrum of 1.

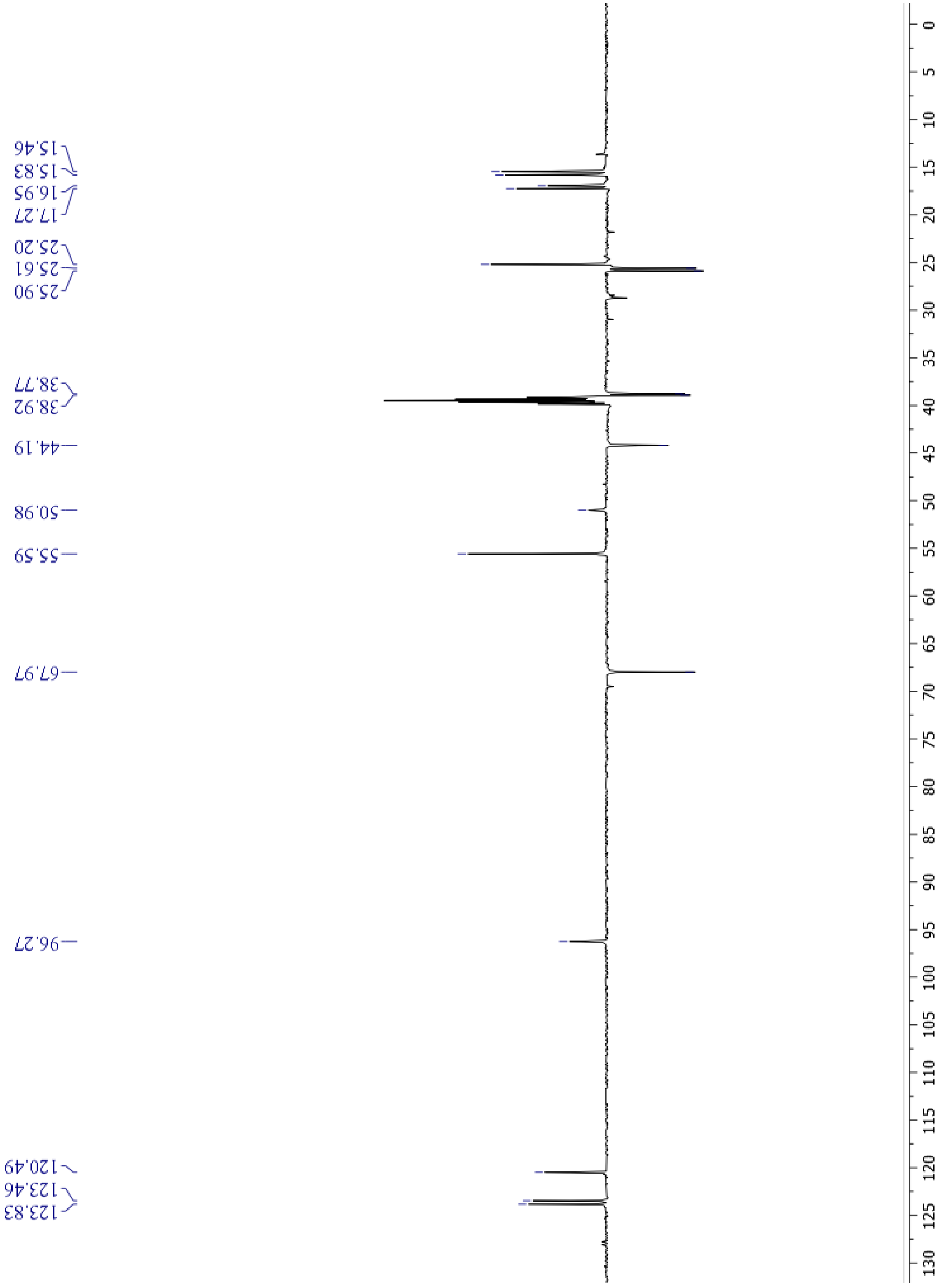

Fig. S3. The DEPT-135 (125 MHz, DMSO-d_6) spectrum of 1.

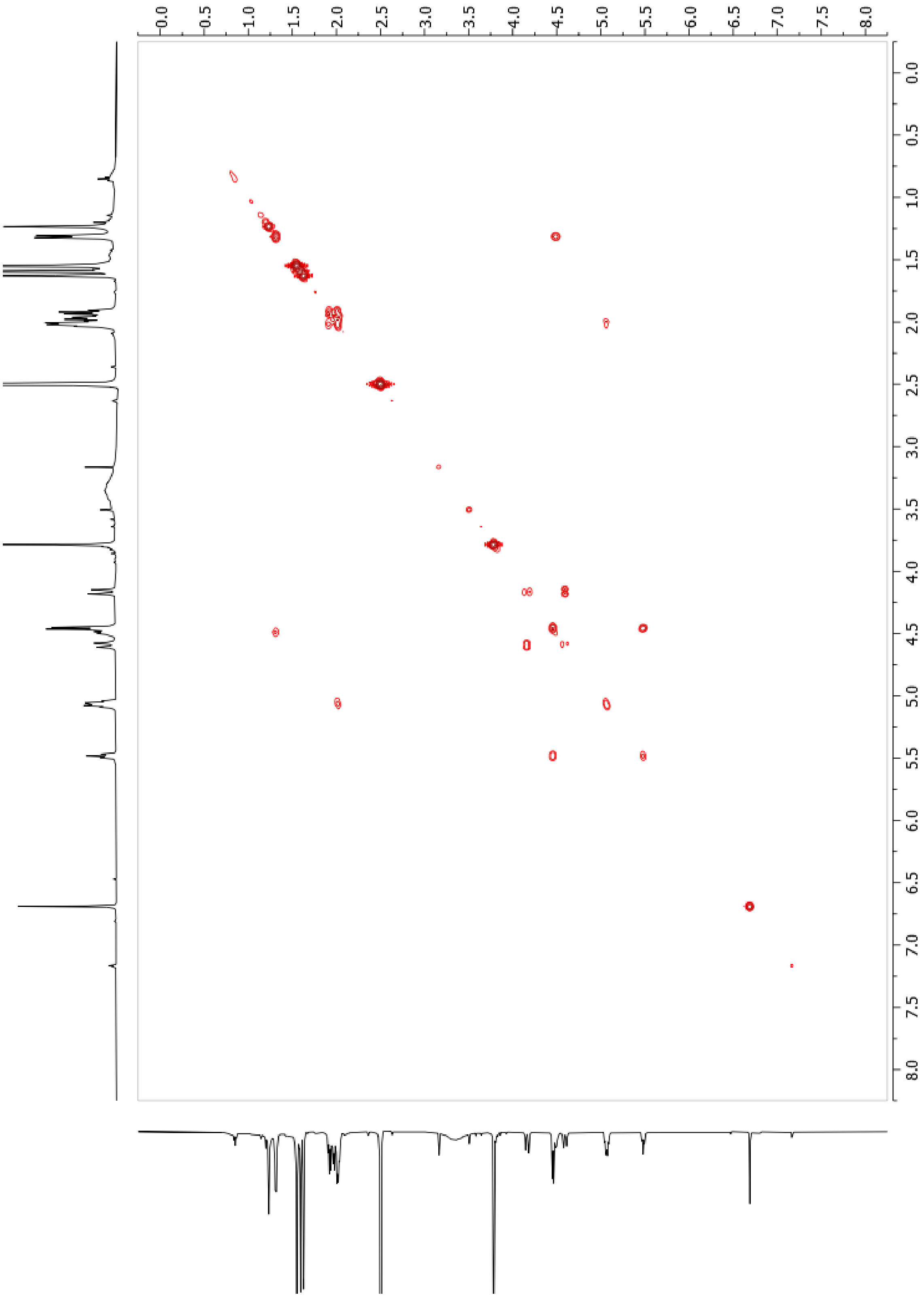

Fig. S4. The COSY (500 MHz, DMSO-d_6) spectrum of 1.

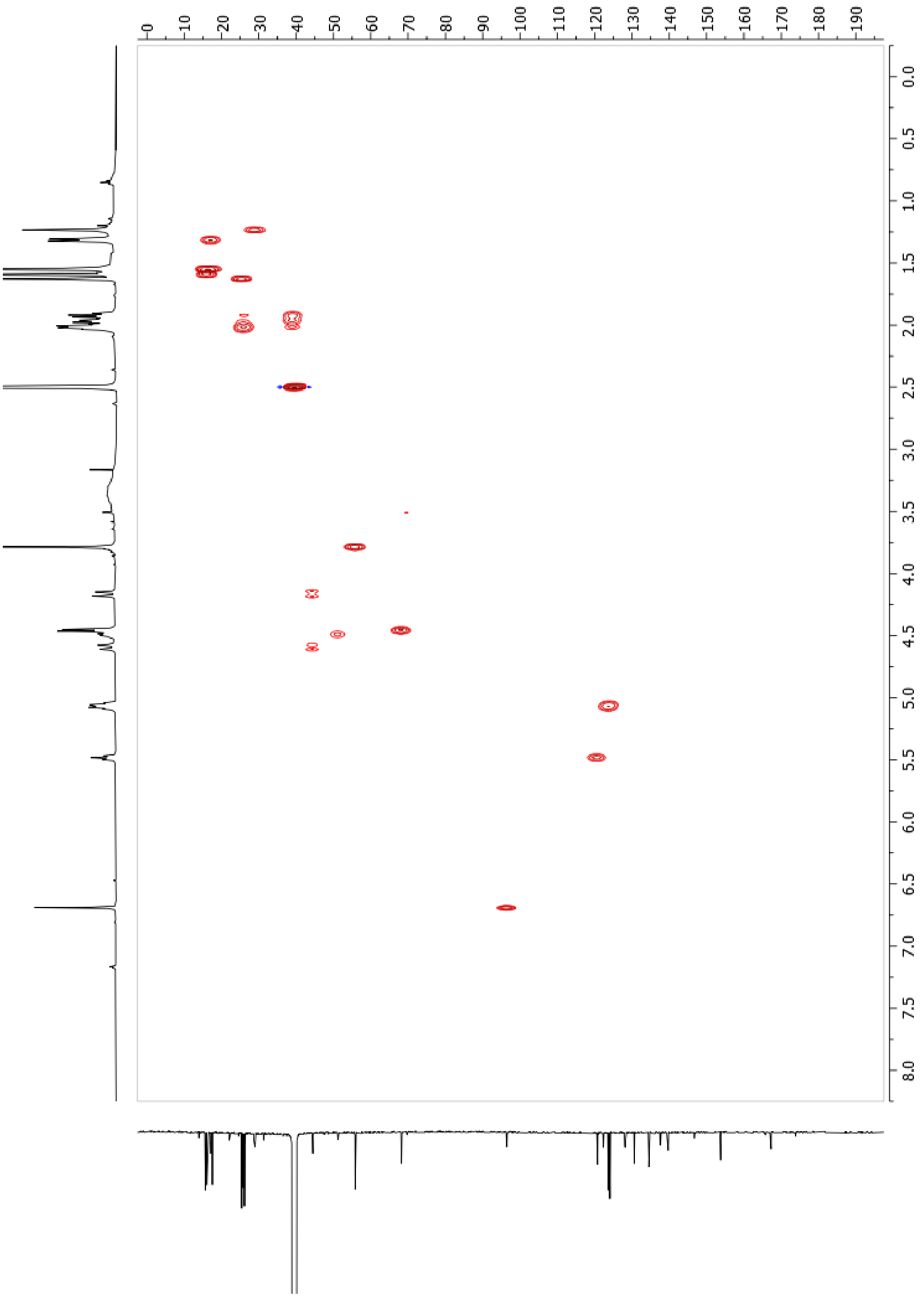

Fig. S5. The HSQC (500 MHz, DMSO-d_6) spectrum of 1.

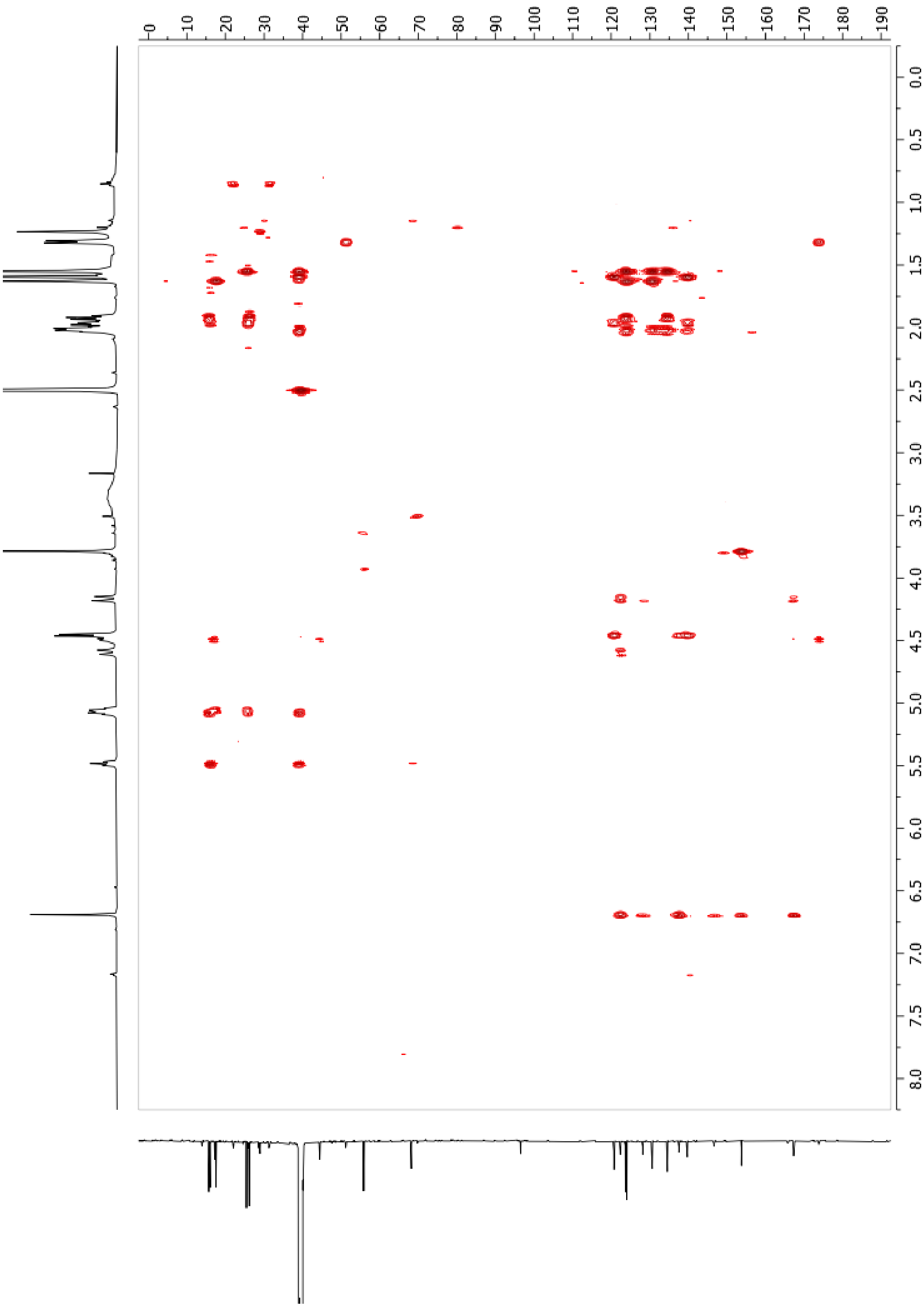

Fig. S6. The HMBC (500 MHz, DMSO-d_6) spectrum of 1.

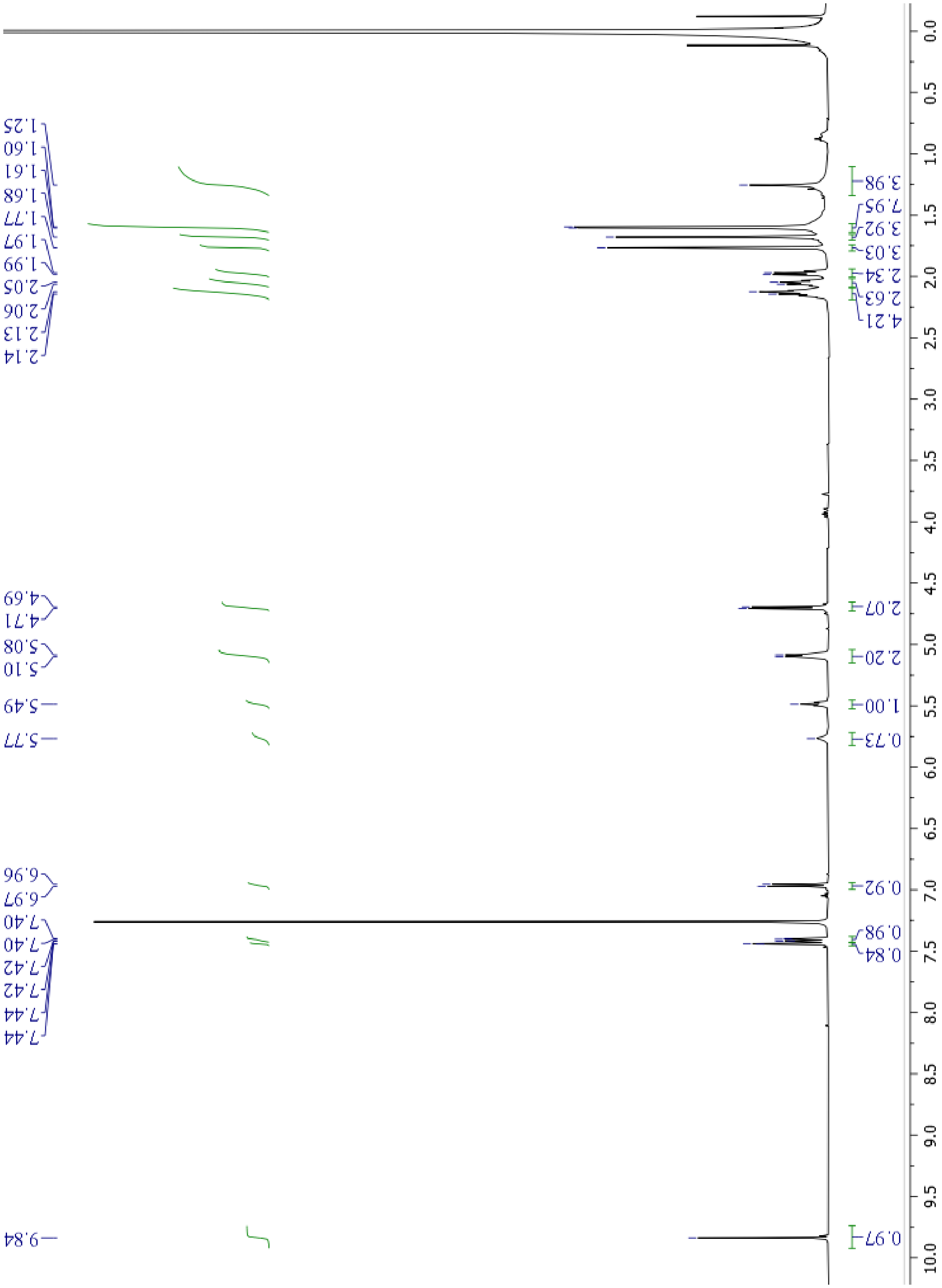

Fig. S7. The ^{1}H NMR (500 MHz, $CDCl_3$) spectrum of 2.

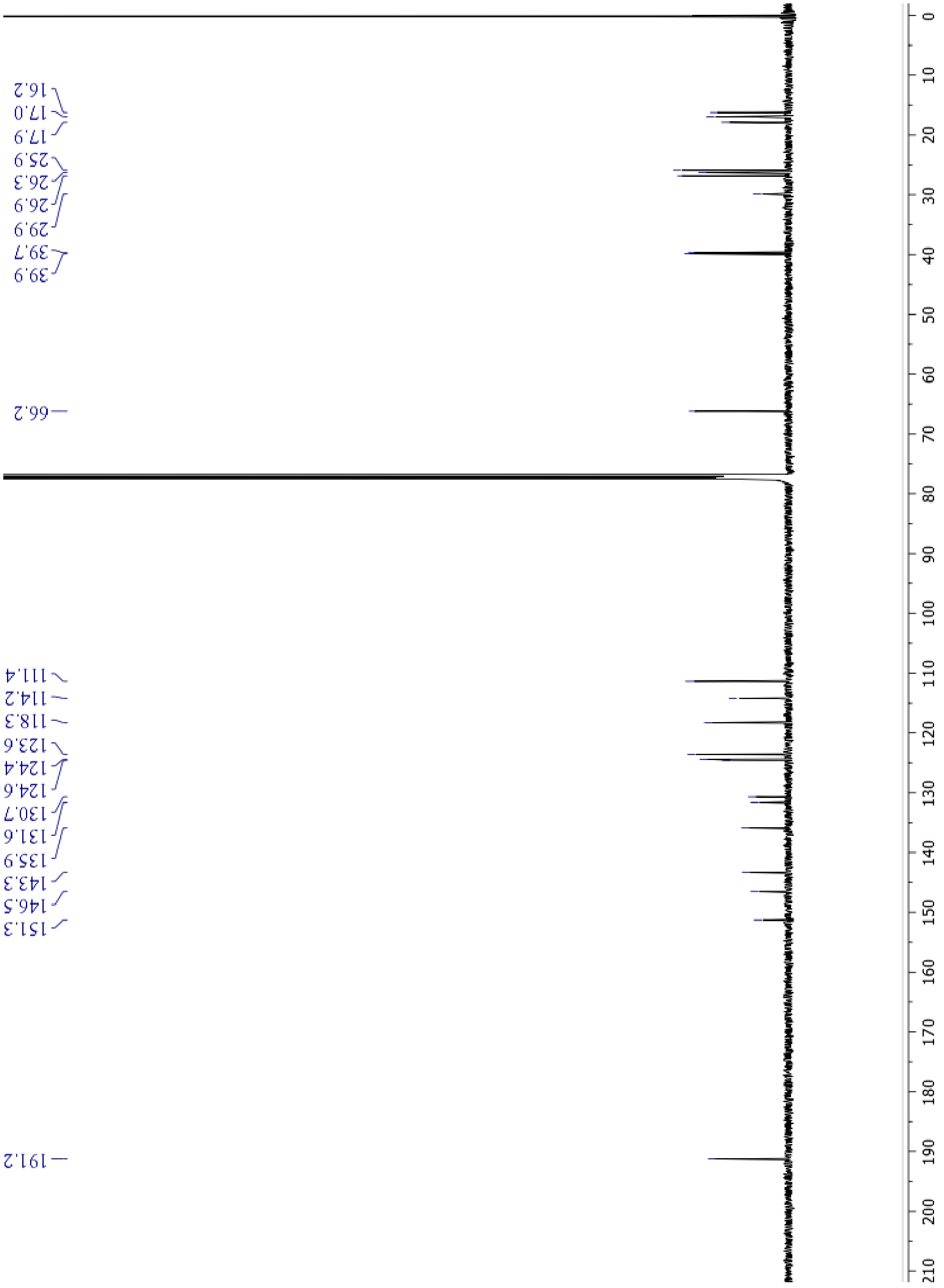

Fig. S8. The ^{13}C NMR (125 MHz, $CDCl_3$) spectrum of 2.

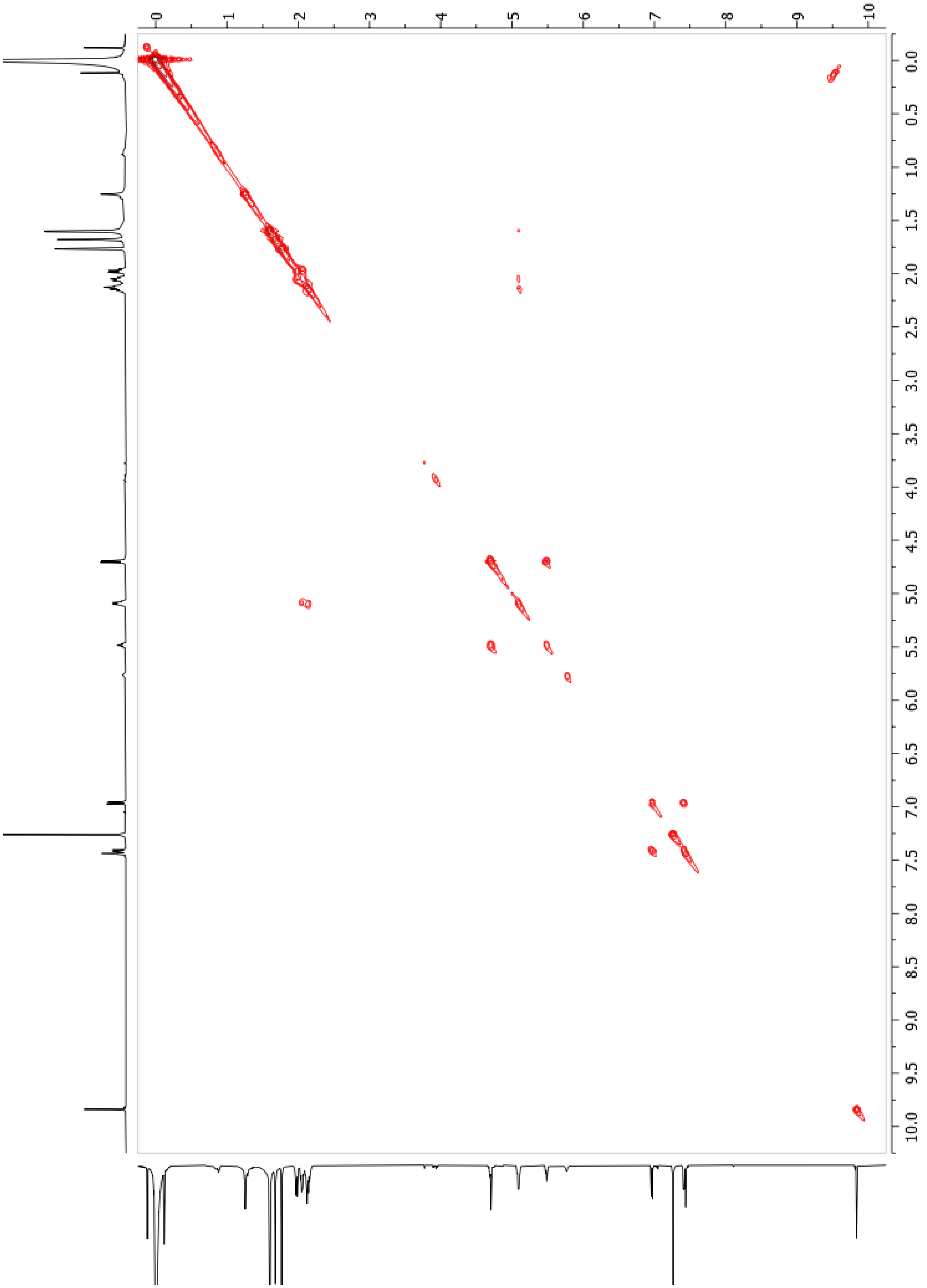

Fig. S9. The COSY (500 MHz, $CDCl_3$) spectrum of 2.

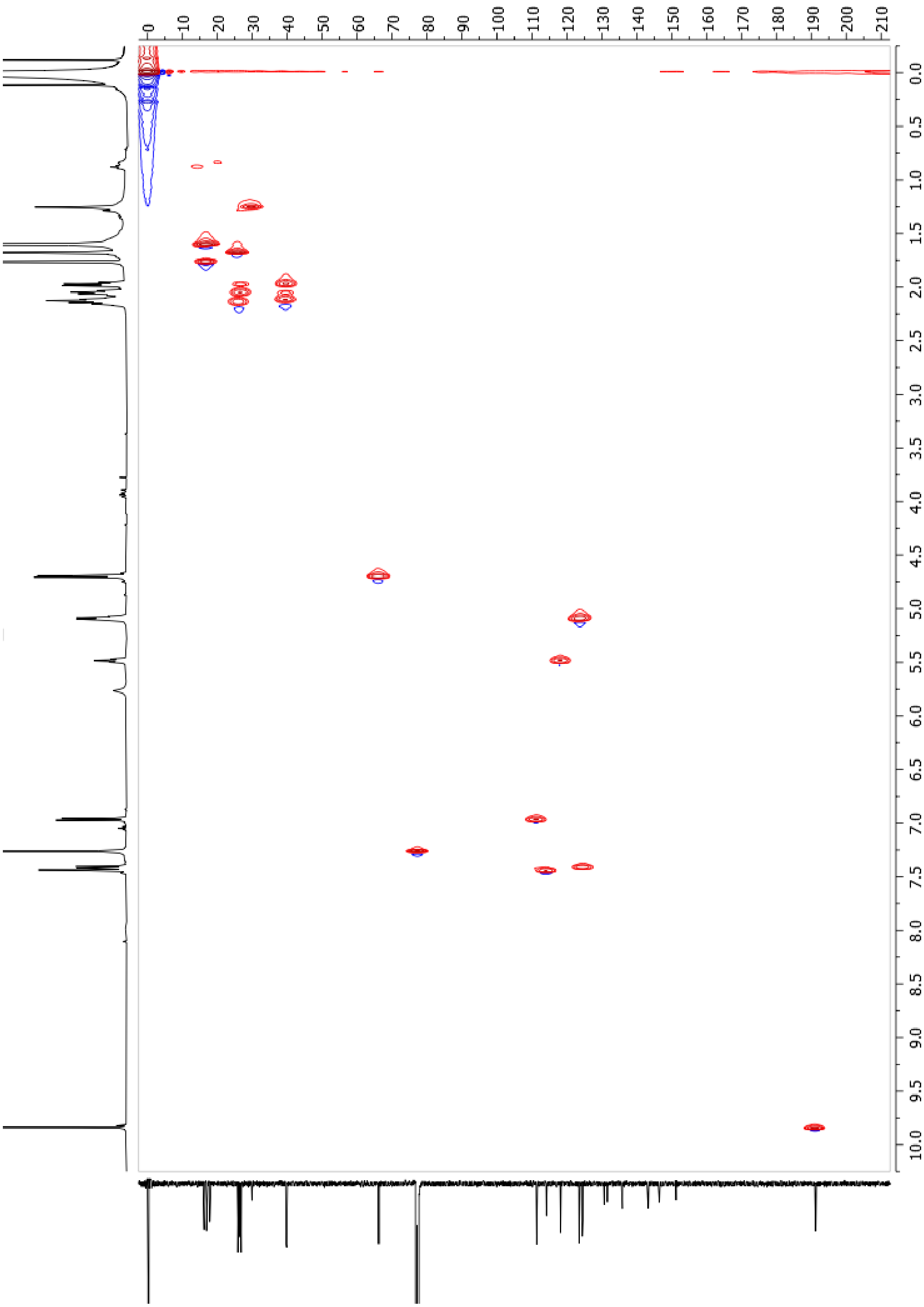

Fig. S10. The HSQC (500 MHz, $CDCl_3$) spectrum of 2.

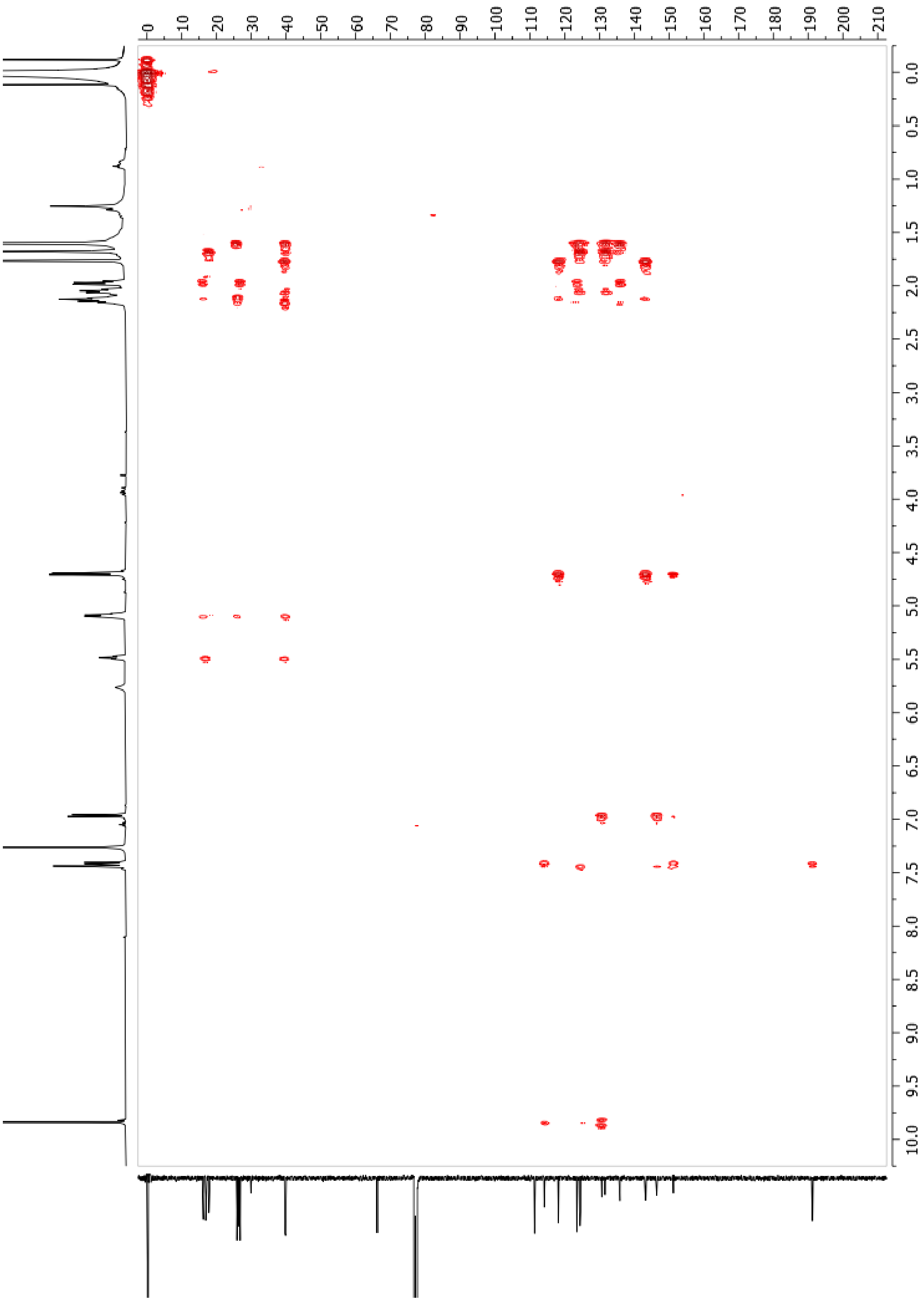

Fig. S11. The HMBC (500 MHz, $CDCl_3$) spectrum of 2.

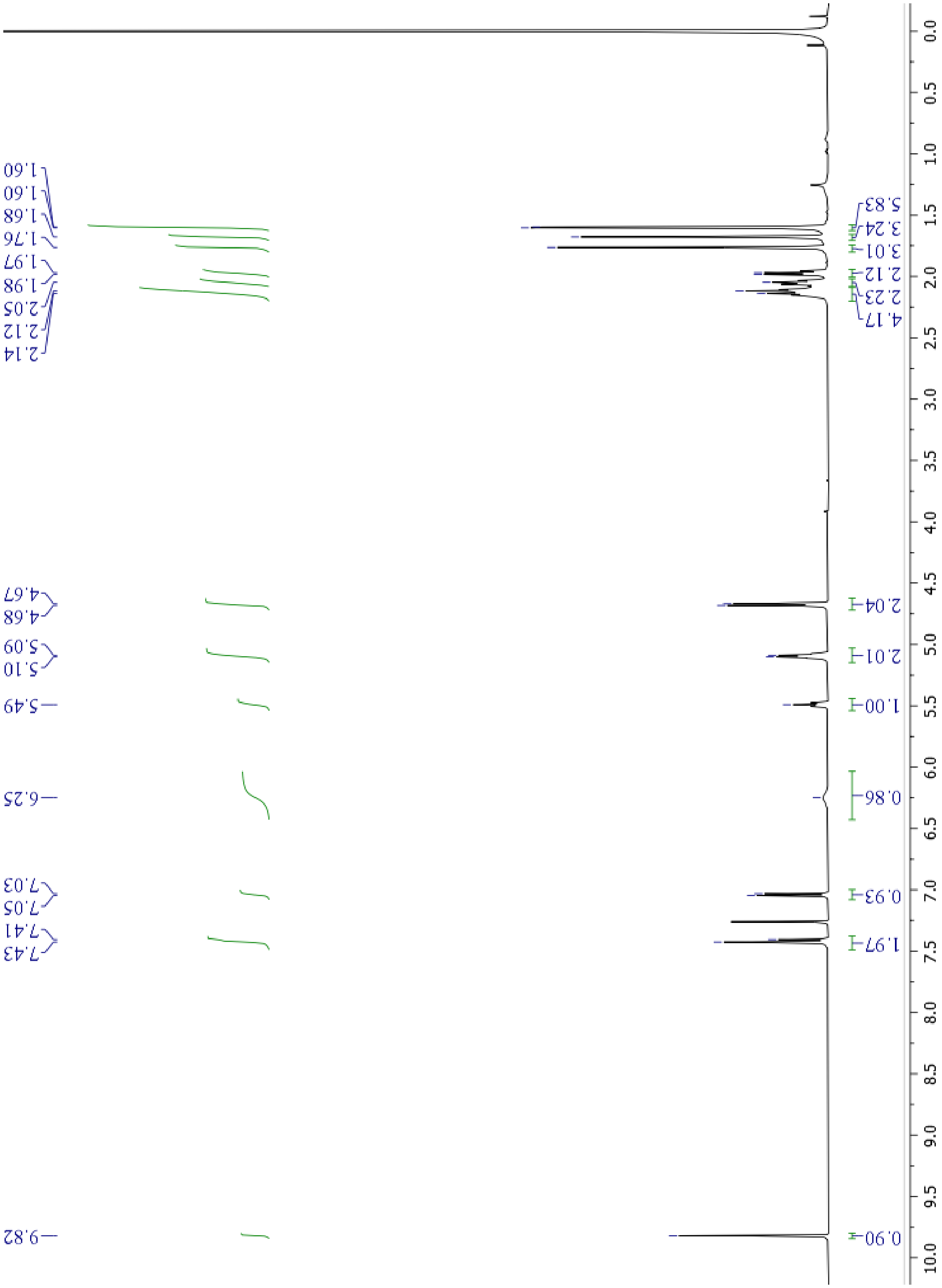

Fig. S12. The ^{1}H NMR (500 MHz, $CDCl_3$) spectrum of 3.

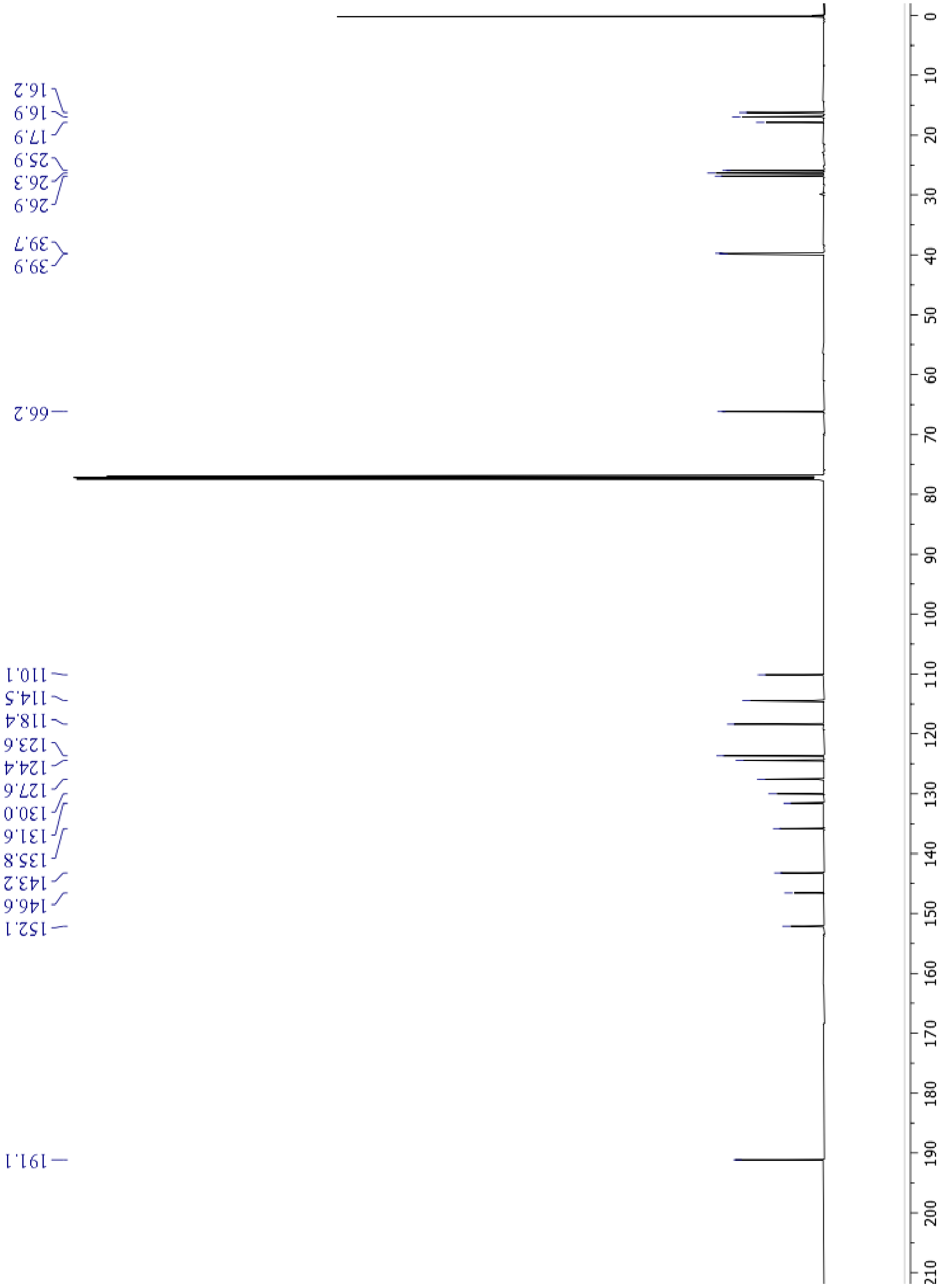

Fig. S13. The ^{13}C NMR (125 MHz, $CDCl_3$) spectrum of 3.

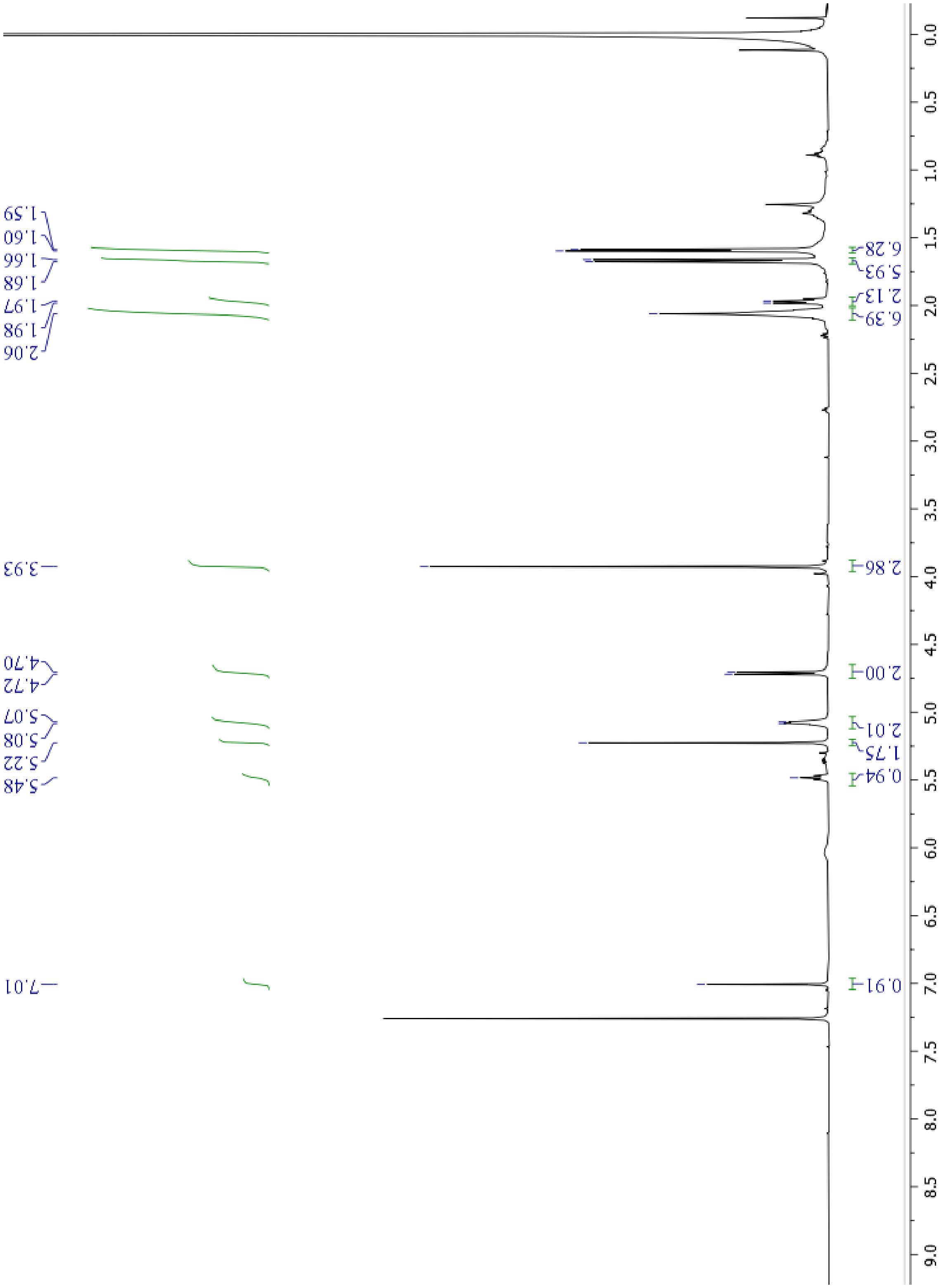

Fig. S14. The ^{1}H NMR (500 MHz, $CDCl_3$) spectrum of 4.

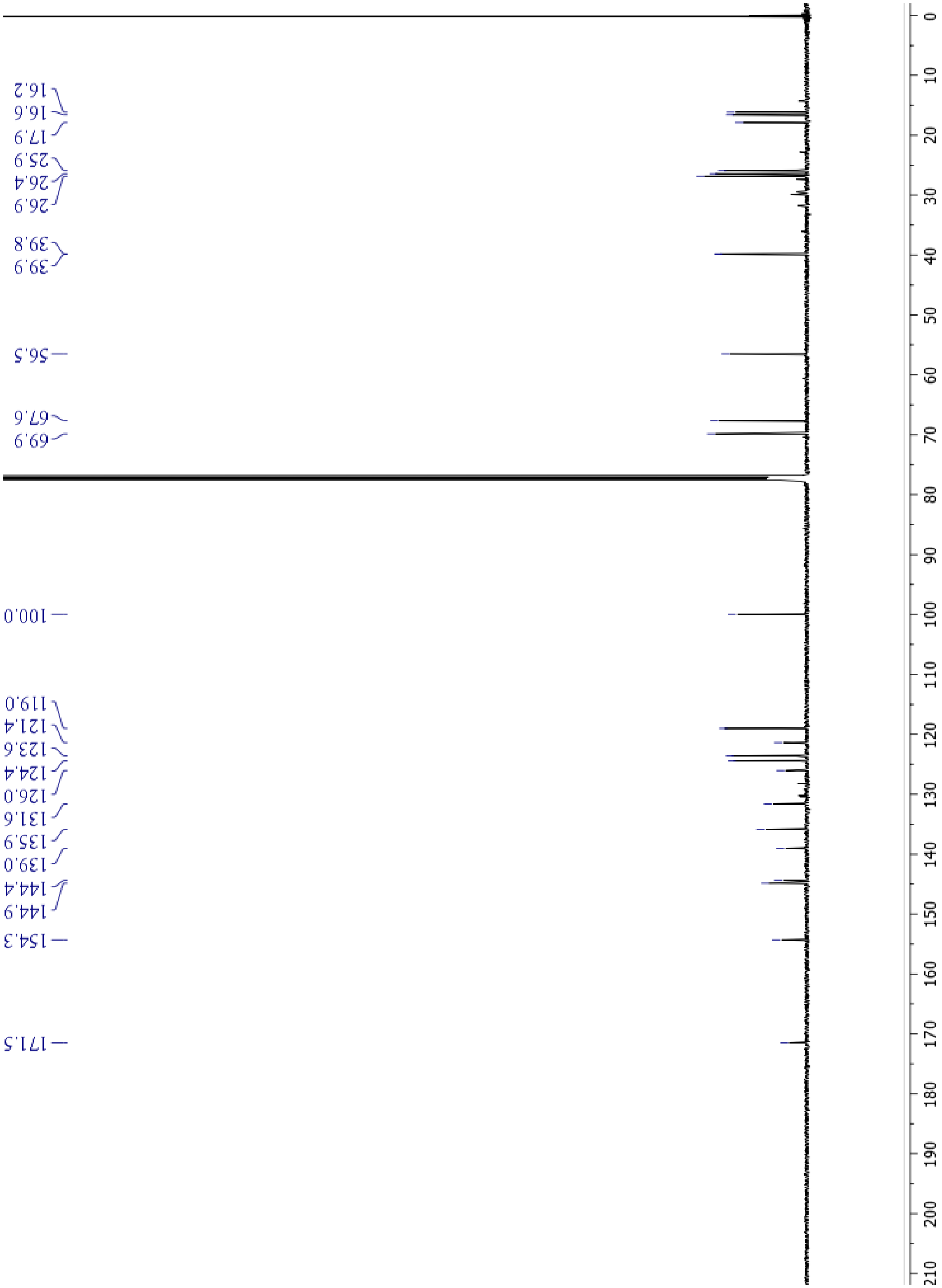

Fig. S15. The ^{13}C NMR (125 MHz, $CDCl_3$) spectrum of 4.

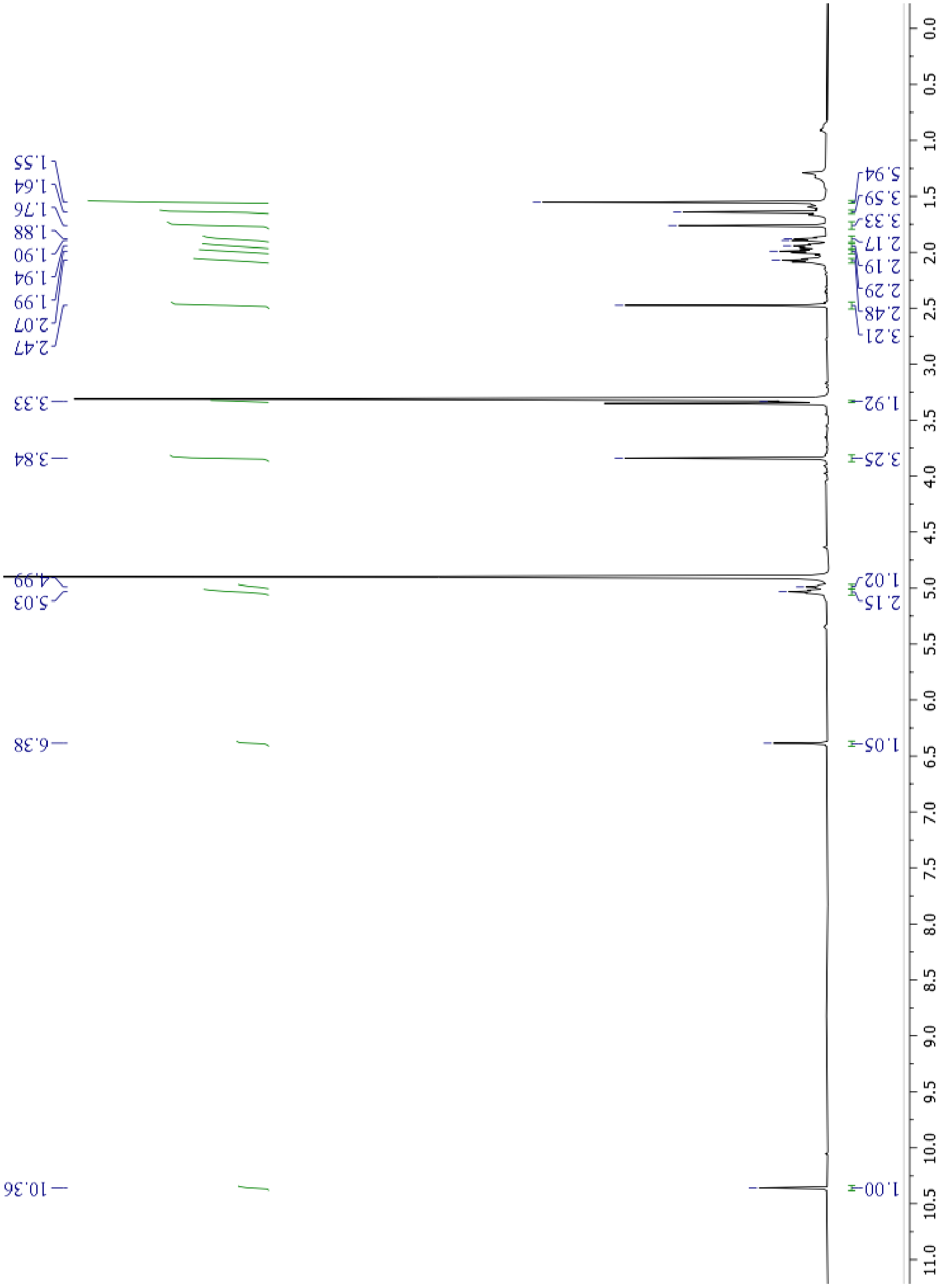

Fig. S16. The ^{1}H NMR (500 MHz, Methanol-d_4) spectrum of 5.

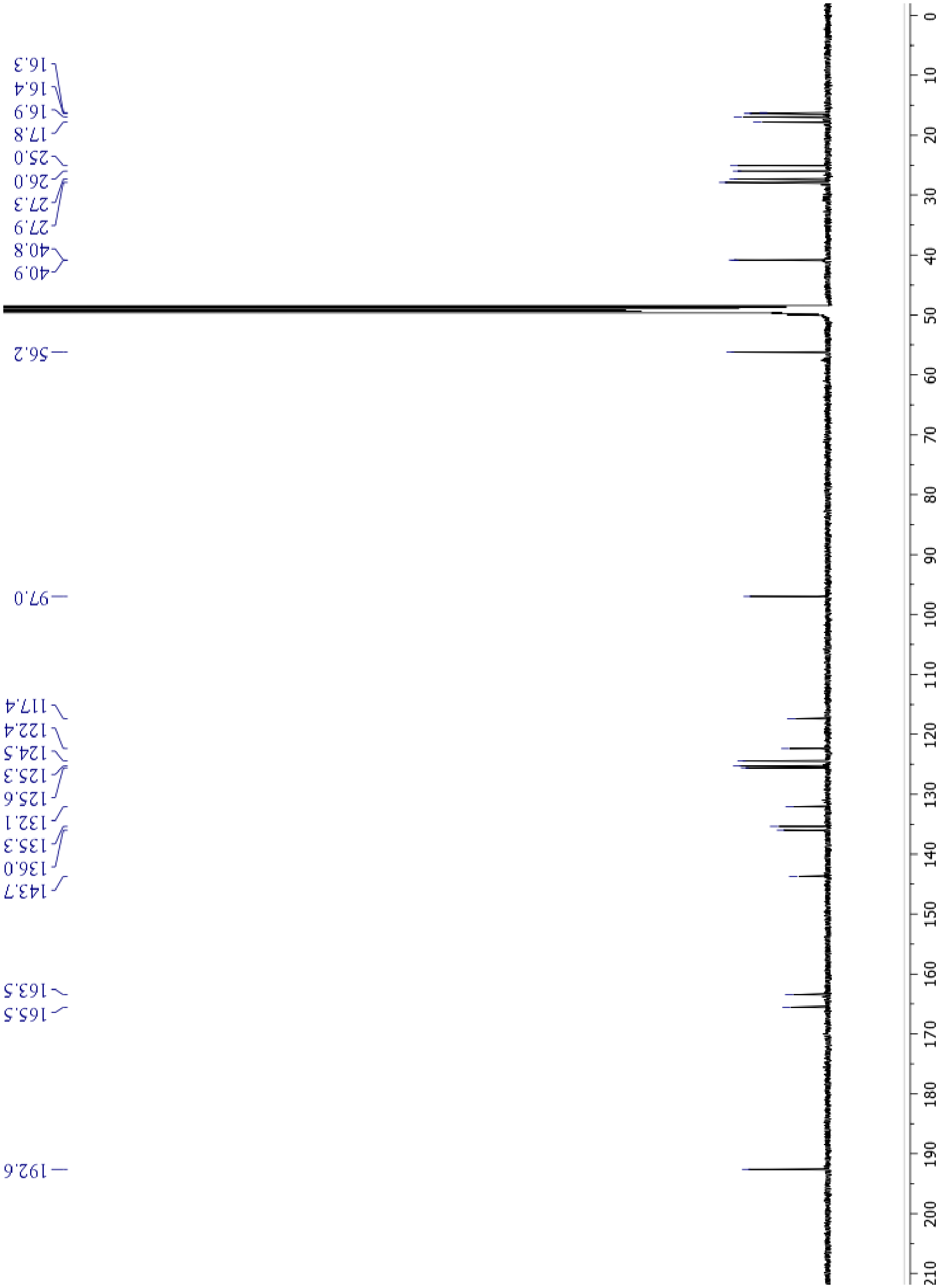

Fig. S17. The ^{13}C NMR (125 MHz, Methanol-*d*$_4$) spectrum of 5.

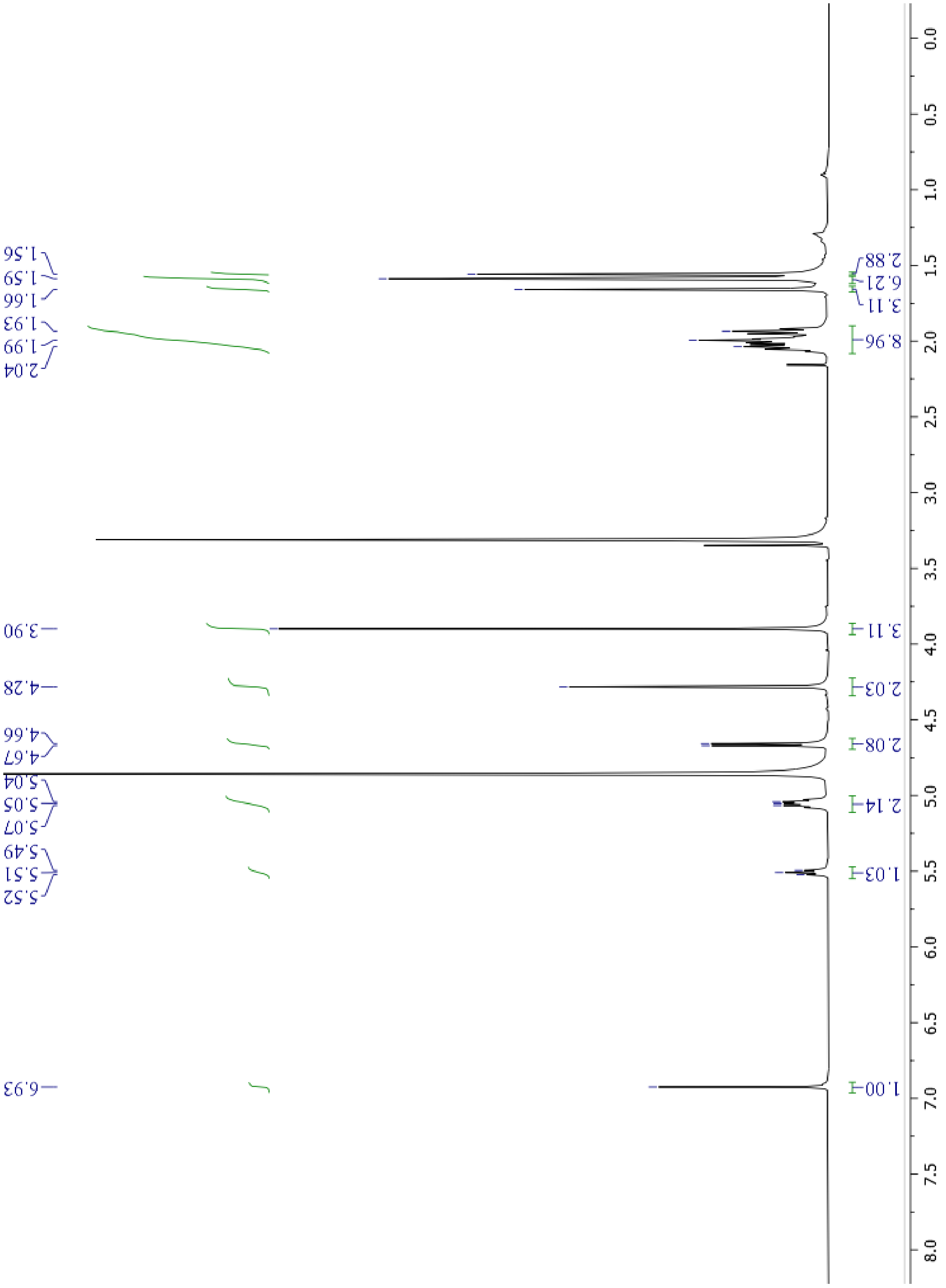

Fig. S18. The ^{1}H NMR (500 MHz, Methanol-d_4) spectrum of 6.

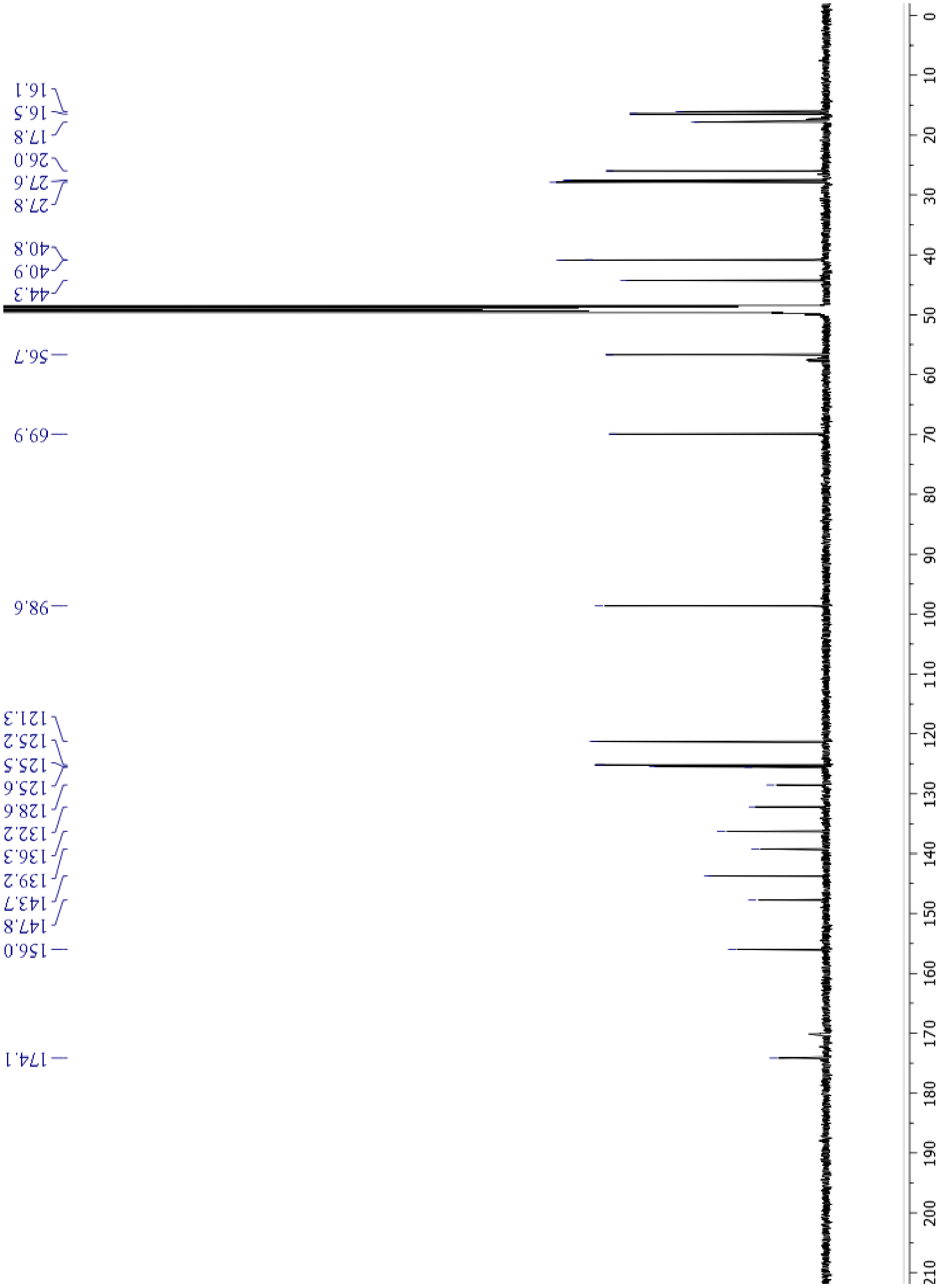

Fig. S19. The ^{13}C NMR (125 MHz, Methanol-d_4) spectrum of 6.

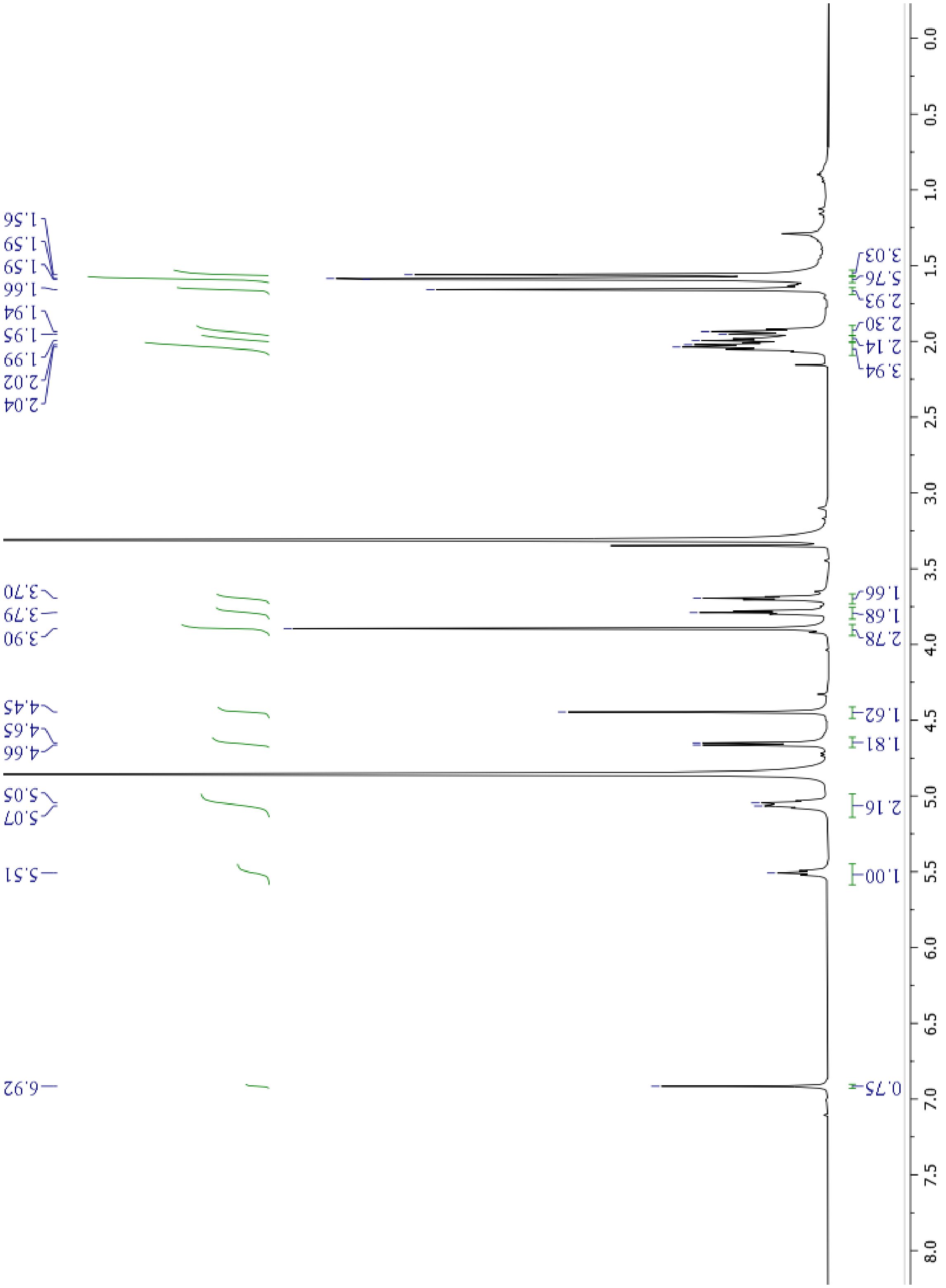

Fig. S20. The ^{1}H NMR (500 MHz, Methanol-d_4) spectrum of 7.

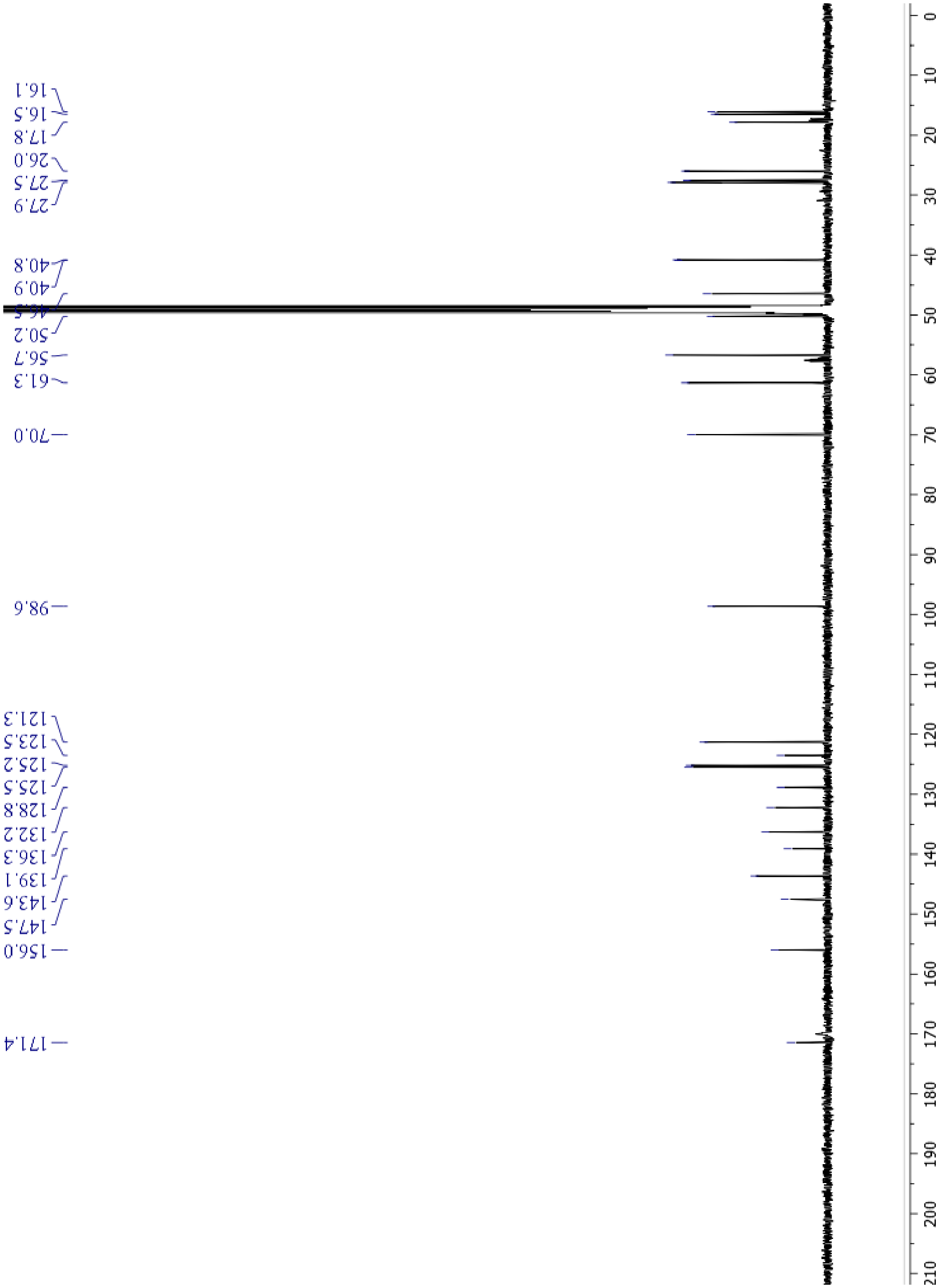

Fig. S21. The ^{13}C NMR (125 MHz, Methanol-d_4) spectrum of 7.

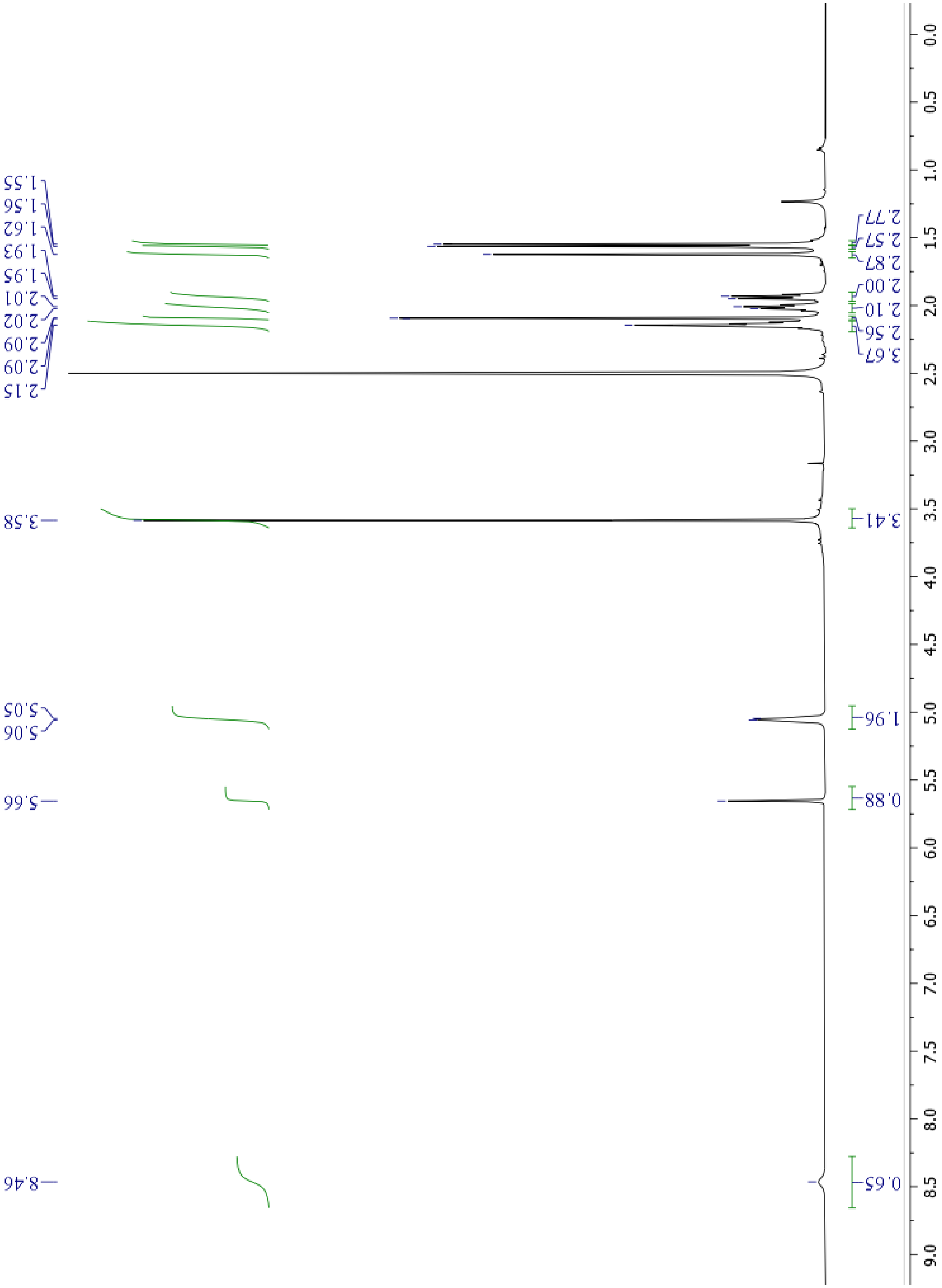

Fig. S22. The ^{1}H NMR (500 MHz, DMSO-d_6) spectrum of 8.

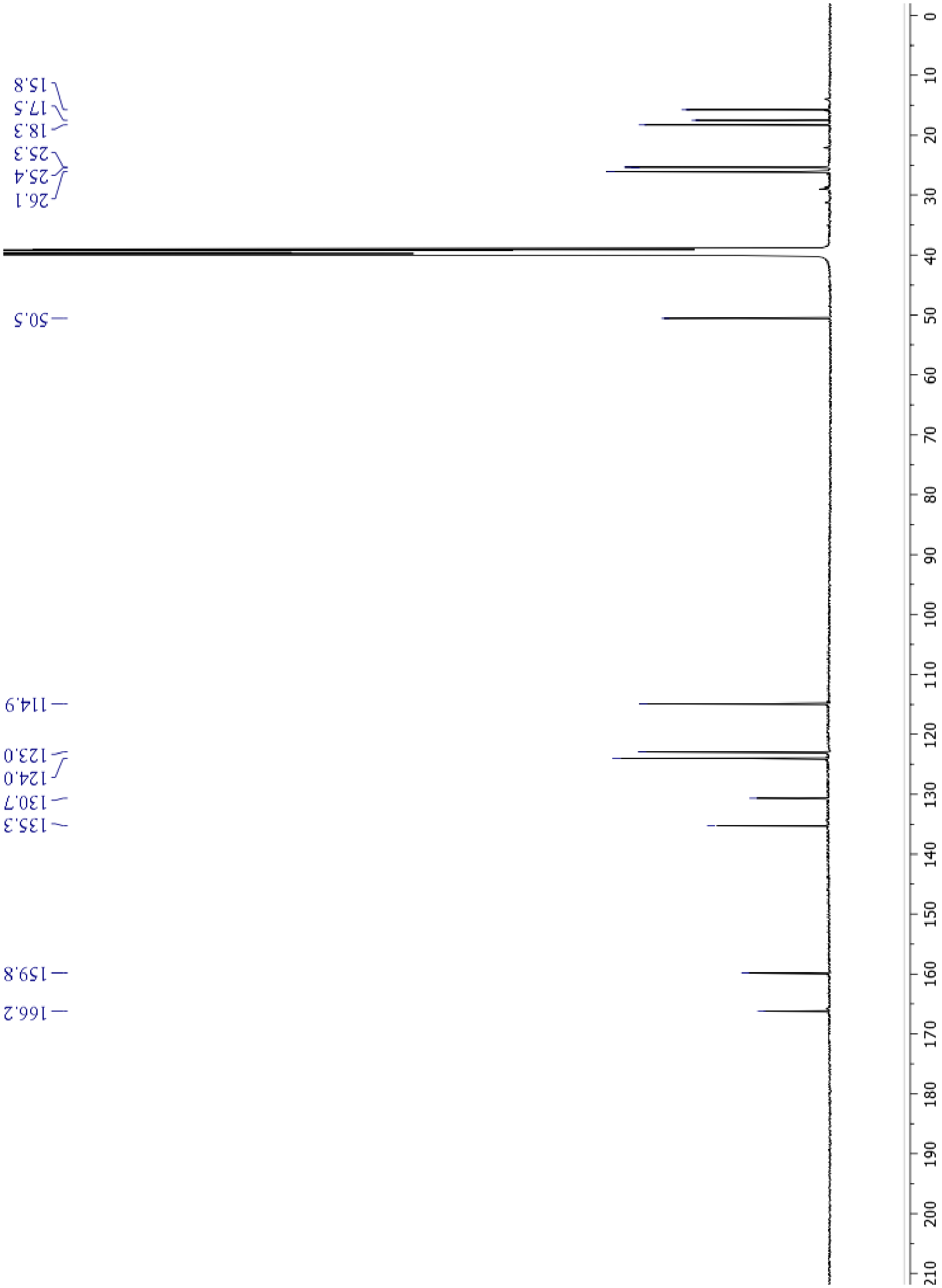

Fig. S23. The ^{13}C NMR (125 MHz, DMSO-d_6) spectrum of 8.

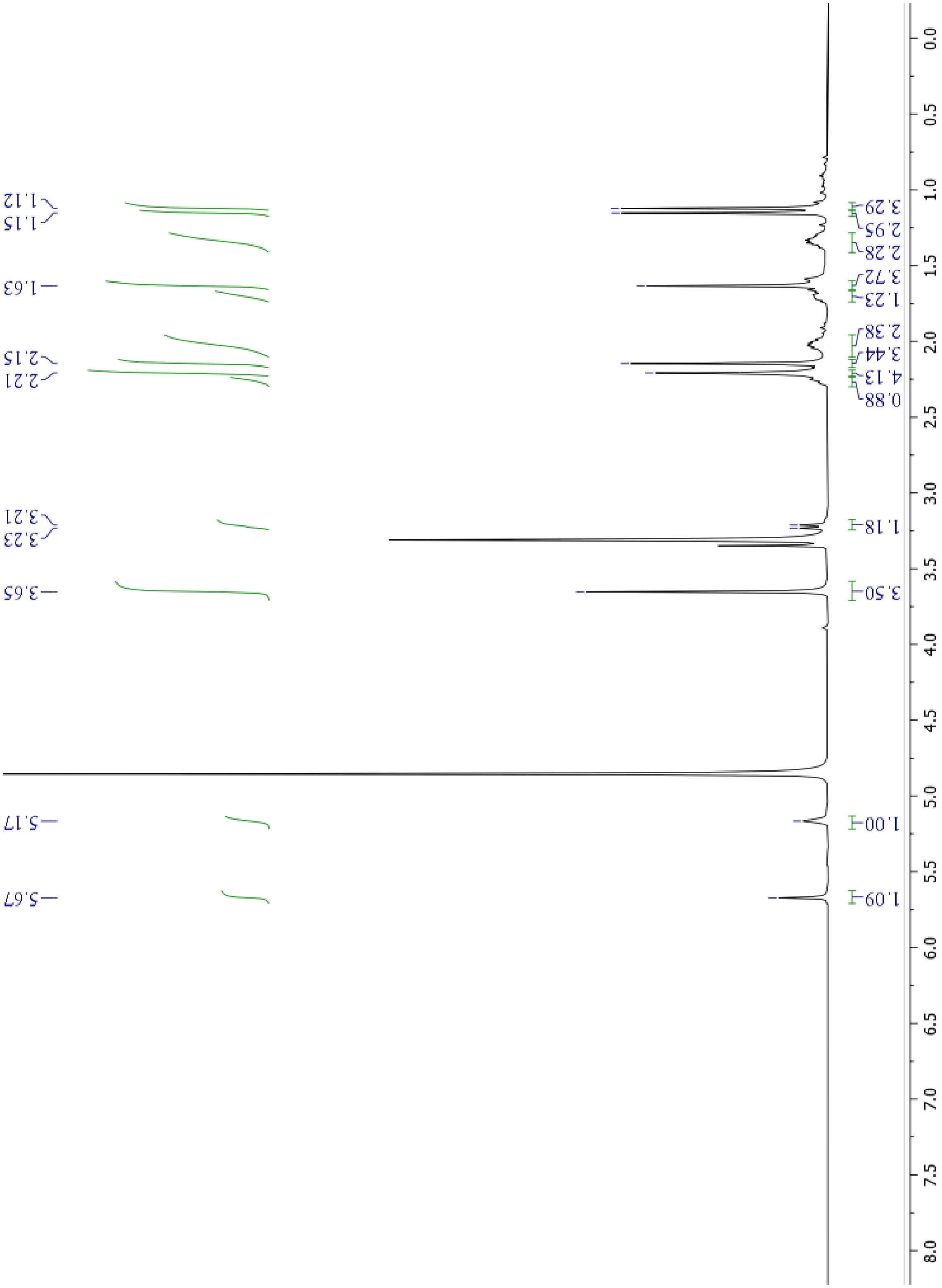

Fig. S24. The ^{1}H NMR (500 MHz, Methanol-d_4) spectrum of 9.

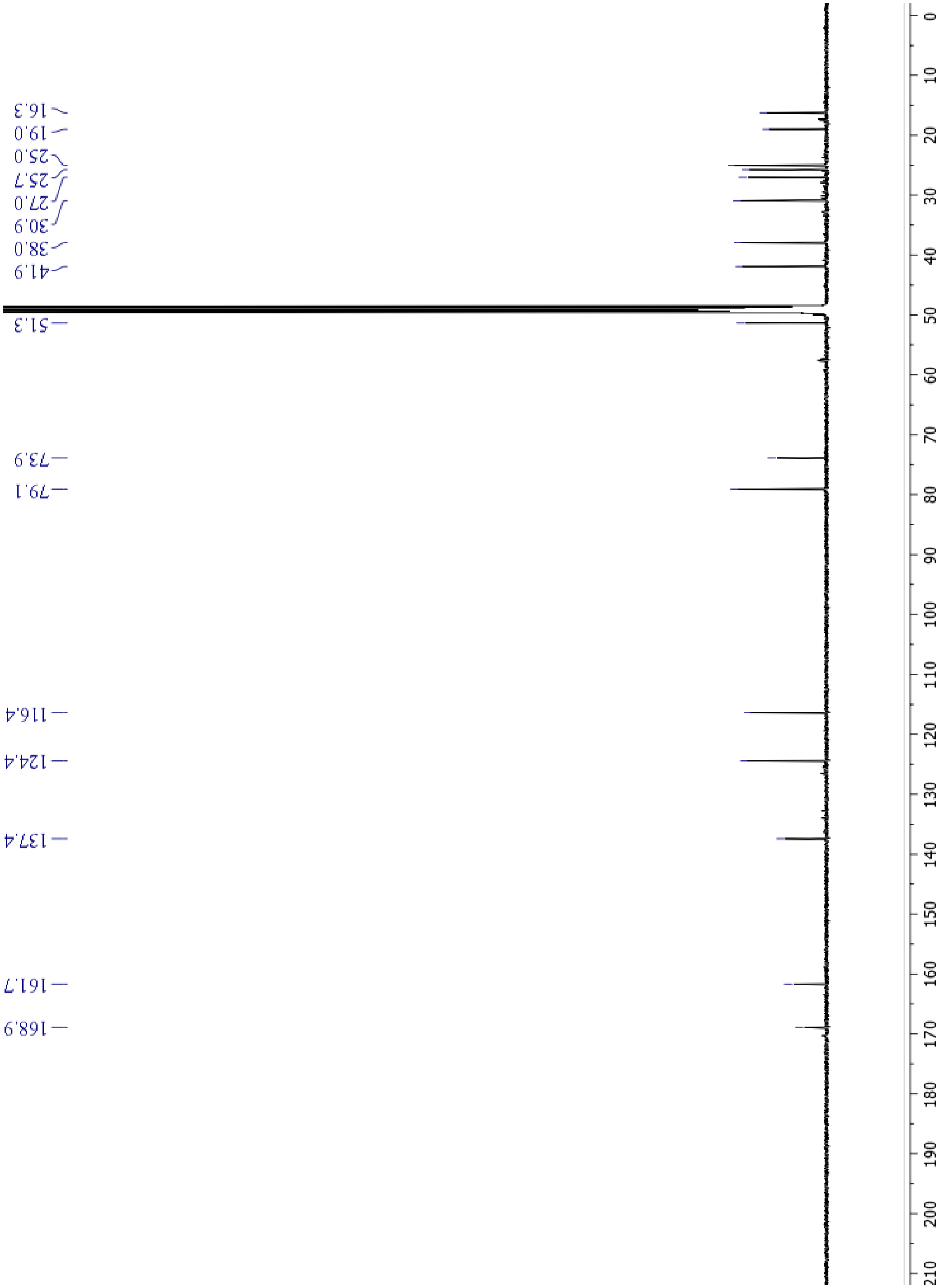

Fig. S25. The ^{13}C NMR (125 MHz, Methanol-*d*$_4$) spectrum of 9.

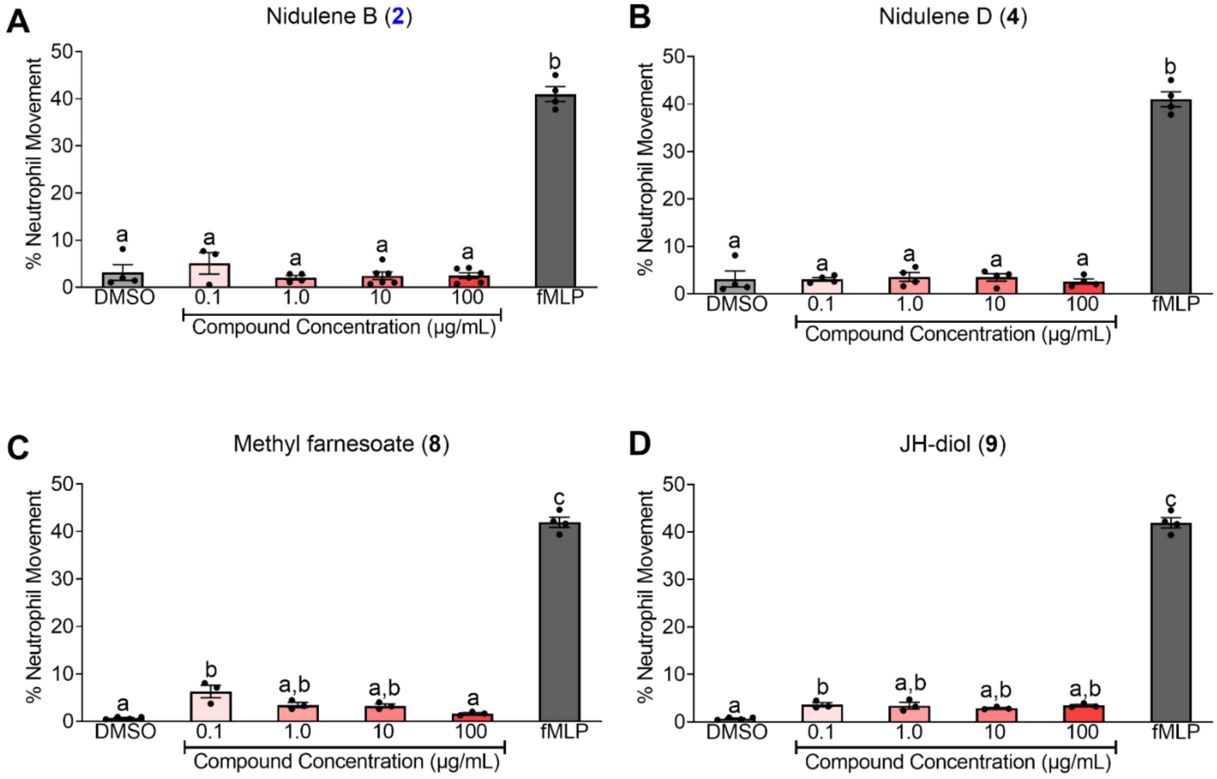

Fig. S26. Human neutrophil chemotaxis. Using a Boyden chamber, neutrophils stained with calcein AM were seeded in the upper chamber at 5 x 10^4 cells/well and incubated at 37 °C for 1 h with the vehicle (DMSO; light grey bar), positive control (fMLP, 10 nM; dark grey bar) or the respective compounds (**2** (**A**)**, 4** (**B**), **8** (**C**), **9** (**D**)) at four different concentrations (0.1, 1, 10, or 100 µg/mL; red bars) found in the lower chamber. Cells that migrated into the lower chamber were quantified using flow cytometry and the percent of live cells that migrated were determined by calculating the percent of live neutrophils from the loading control. Samples are listed as average ± SEM. Using a one-way ANOVA, samples were assessed and letters are used to represent statistical differences.

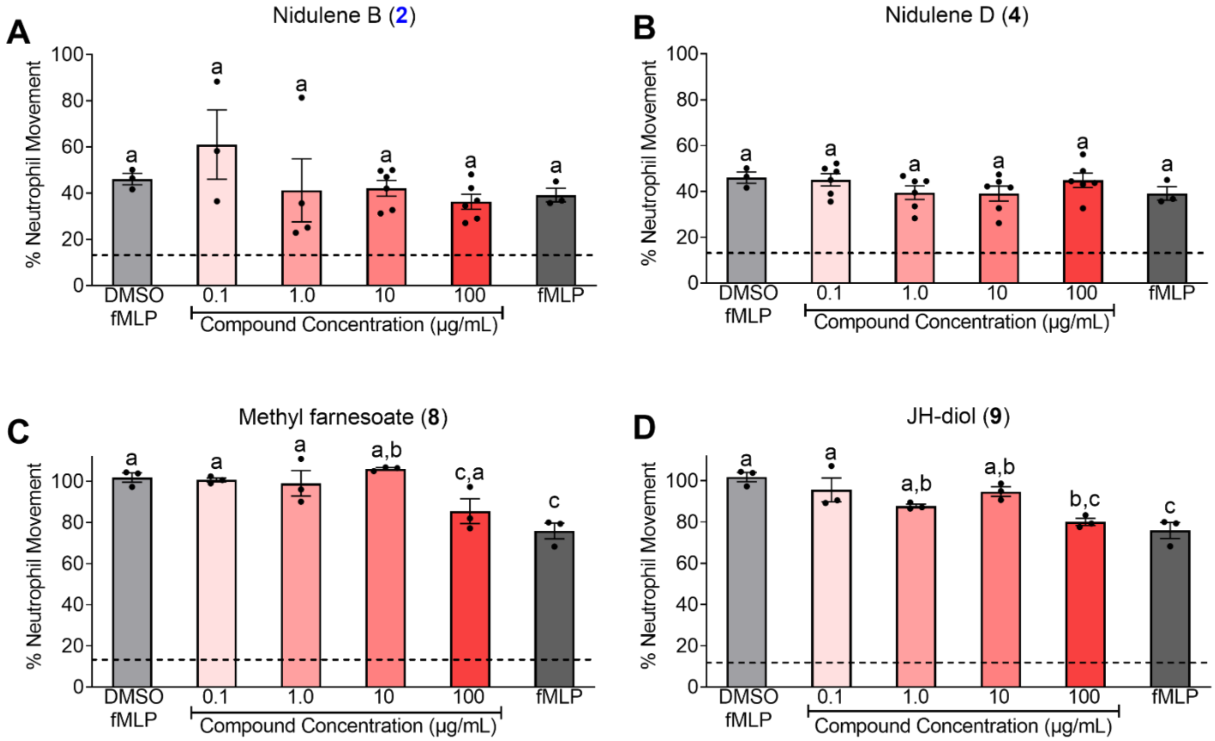

Fig. S27. Inhibition of fMLP-driven neutrophil chemotaxis. Using a Boyden chamber, neutrophils stained with calcein AM were seeded in the upper chamber at 5 x 10^4 cells/well and incubated at 37 °C for 1 h with the vehicle (DMSO; dotted line), the DMSO vehicle with fMLP (fMLP at 10 nM; light grey bar), positive control (fMLP, 10 nM; dark grey bar) or the respective compounds **2** (**A**), **4** (**B**), **8** (**C**), or **9** (**D**) at four different concentrations (0.1, 1, 10, or 100 µg/mL; red bars) found in the lower chamber. Cells that migrated into the lower chamber were quantified using flow cytometry and the percent of live cells that migrated was determined by calculating the percent live of neutrophils from the loading control. Samples are listed as average ± SEM. Compounds **2** and **4** were tested at a different time than compounds **8** and **9** accounting for differences in controls. Using a one-way ANOVA, samples were assessed and letters are used to represent statistical differences.

Table S1. Bacterial and fungal strains.

**Strain**	**Strain name (genotype)**	**Reference**
Pseudogymnoascus destructans	20631-21	Drees, *et al.* (*55*) (ATCC#: MYA-4855)
Aspergillus nidulans	RJW256 (*pyrG89, pyroA4, ΔST::afpyrG, veA1*)	Bok, *et al.* (*11*)
Aspergillus nidulans	TJW167 (*pyrG89, pyroA4, ΔST::afpyrG, BACpyrG::AMA1, veA1*)	Bok, *et al.* (*11*)
Aspergillus nidulans	TJW336 PdFAC1	this work (GenBank#: OR972369)
Aspergillus nidulans	TJW337 PdFAC1Δ*sqsA*	this work
Escherichia coli	igMax™ DH10B strain	Intact Genomics
Escherichia coli	SW101 ΔgalK	Warming *et al.* (*51*)

Table S2. Structure activity relationship (SAR) summary

#	Name	Structure	Human neutrophil chemotaxis
**1**	Nidulene A	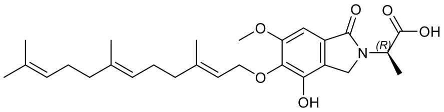	Inhibited at 100 μg/mL
**2**	Nidulene B	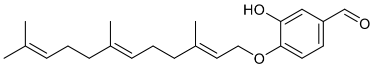	ND
**3**	Nidulene C	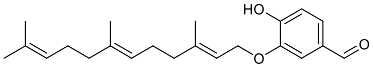	Inhibited at 100 μg/mL
**4**	Nidulene D	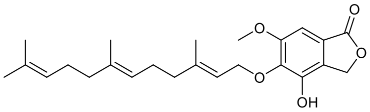	ND
**5**	Nidulene E	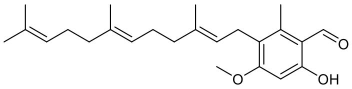	Induced at 100 μg/mL
**6**	Aspernidine A	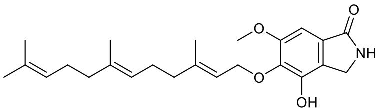	Cytotoxic at 100 μg/mL
**7**	Aspernidine F	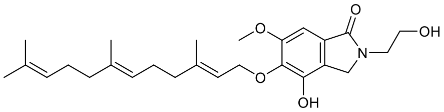	Inhibited at 100 μg/mL
**8**	Methyl farnesoate	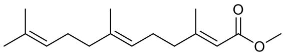	ND
**9**	JH-diol	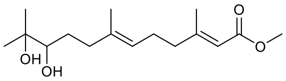	ND

ND represents that no significant result was detected.

Table S3. Vectors and primers

**Vectors**	**Reference**
PdFAC1P6	this study
PdFAC1P6Δ*sqsA*	this study

**Primer**	**Sequence**	**Reference**
PdFAC1-807Kan-F	TTCACCTCAATCcgacctgcag	
PdFAC1-807Kan-R	TAACCCGGAAAGgtcgaggctg	
	ttcacctcaatccgacctgcag	
PdFAC1 confirm primers		
Pd1F	acatgaccattccgttggagg	this study
Pd1R	aaggtagacggtgtctgtcg	this study
PdFAC1Δsqs confirm primers		
Pd1DsqsF	ttcacctcaatccgacctgcag	this study
Pd1DsqsR	taacccggaaaggtcgaggctg	this study
Deletion primers*		
PdFAC1P6SqSdel-f	Caaaccagacaccatttactctccggcgacatt<br>ccgccttcacctcaatccgacctgcagcctgttga	this study
PdFAC1P6SqSdel-r	Ccccgcaatagagtatataaatcgtcctaaagtt<br>gggtgtaacgtagttagtcgaggctgatcagcga	this study

* Primer sequences underlined shows 50-bp homologous for targeted gene deletion.

Table S4. Annotation of 26 orfs from PdFAC1.

ORF	JGI Gene ID	Predicted Function/Domains
26	GMDG_02820	endonuclease
25	GMDG_02819	Aldehyde dehydrogenase
24	GMDG_02818	Acyl-CoA synthetase
23	GMDG_02817	integral membrane protein
22	GMDG_02816	signal recognition particle protein
21	GMDG_02815	exonuclease
20	GMDG_02814	IFRD domain (growth/differentiation)
19	GMDG_02813	unknown
18	GMDG_02812	translation initiation factor
17	GMDG_02811	AIM24 domain (mitochondria biogenesis)
16	GMDG_02810	unc-13 homolog (vesicle trafficking)
15	GMDG_02809	mediator complex (transcription regulation)
14	GMDG_02808	bromodomain
13	GMDG_02807	**farnesyl-diphosphate farnesyl transferase/squalene synthase**
12	GMDG_02806	unknown
11	GMDG_02805	glycyl-tRNA synthetase or TonB-dependent receptor
10	GMDG_02804	Unknown (maybe related to mRNA processing)
9	GMDG_02803	Unknown (maybe related to cytoskeleton)
8	GMDG_02802	WD40 repeat
7	GMDG_02801	metallo-dependent hydrolase/deaminase
6	GMDG_02800	exocyst complex protein (vesicle trafficking)
5	GMDG_02799	universal stress protein
4	GMDG_02798	DnaJ (chaperone protein)
3	GMDG_02797	unknown
2	GMDG_02796	cytochrome P450
1	GMDG_02795	3,4-dihydroxy-2-butanone 4-phosphate synthase

REFERENCES AND NOTES

1. N. P. Keller, Fungal secondary metabolism: Regulation, function and drug discovery. *Nat. Rev. Microbiol.* **17**, 167–180 (2019).

2. A. A. Brakhage, Regulation of fungal secondary metabolism. *Nat. Rev. Microbiol.* **11**, 21–32 (2013).

3. S. Sanchez, A. L. Demain, Bioactive products from fungi. *Food Bioact.* 59–87 (2017).

4. Y. Matsuda, I. Abe, Biosynthesis of fungal meroterpenoids. *Nat. Prod. Rep.* **33**, 26–53 (2016).

5. R. Geris, T. J. Simpson, Meroterpenoids produced by fungi. *Nat. Prod. Rep.* **26**, 1063–1094 (2009).

6. M. Nazir, M. Saleem, M. I. Tousif, M. A. Anwar, F. Surup, I. Ali, D. Wang, N. Z. Mamadalieva, E. Alshammari, M. L. Ashour, A. M. Ashour, I. Ahmed, Elizbit, I. R. Green, H. Hussain, Meroterpenoids: A comprehensive update insight on structural diversity and biology. *Biomolecules* **11**, 957 (2021).

7. M. Zhao, Y. Tang, J. Xie, Z. Zhao, H. Cui, Meroterpenoids produced by fungi: Occurrence, structural diversity, biological activities, and their molecular targets. *Eur. J. Med. Chem.* **209**, 112860 (2021).

8. M. Jiang, Z. Wu, L. Liu, S. Chen, The chemistry and biology of fungal meroterpenoids (2009-2019). *Org. Biomol. Chem.* **19**, 1644–1704 (2021).

9. A. A. Brakhage, V. Schroeckh, Fungal secondary metabolites—Strategies to activate silent gene clusters. *Fungal Genet. Biol.* **48**, 15–22 (2011).

10. J. W. Bok, R. Ye, K. D. Clevenger, D. Mead, M. Wagner, A. Krerowicz, J. C. Albright, A. W. Goering, P. M. Thomas, N. L. Kelleher, N. P. Keller, C. C. Wu, Fungal artificial chromosomes for mining of the fungal secondary metabolome. *BMC Genomics* **16**, 343 (2015).

11. K. D. Clevenger, J. W. Bok, R. Ye, G. P. Miley, M. H. Verdan, T. Velk, C. Chen, K. Yang, M. T. Robey, P. Gao, M. Lamprecht, P. M. Thomas, M. N. Islam, J. M. Palmer, C. C. Wu, N. P. Keller, N. L. Kelleher, A scalable platform to identify fungal secondary metabolites and their gene clusters. *Nat. Chem. Biol.* **13**, 895–901 (2017).

12. L. K. Caesar, M. T. Robey, M. Swyers, M. N. Islam, R. Ye, P. P. Vagadia, G. E. Schiltz, P. M. Thomas, C. C. Wu, N. L. Kelleher, N. P. Keller, J. W. Bok, Heterologous expression of the unusual terreazepine biosynthetic gene cluster reveals a promising approach for identifying new chemical scaffolds. *mBio* **11**, e01691-20 (2020).

13. G. Pontecorvo, J. A. Roper, L. M. Hemmons, K. D. Macdonald, A. W. J. Bufton, The genetics of *Aspergillus nidulans*. *Adv. Genet.* **5**, 141–238 (1953).

14. Y.-M. Chiang, T.-S. Lin, C. C. C. Wang, Total heterologous biosynthesis of fungal natural products in *Aspergillus nidulans*. *J. Nat. Prod.* **85**, 2484–2518 (2022).

15. L. K. Caesar, N. L. Kelleher, N. P. Keller, In the fungus where it happens: History and future propelling *Aspergillus nidulans* as the archetype of natural products research. *Fungal Genet. Biol.* **144**, 103477 (2020).

16. M. T. Drott, R. W. Bastos, A. Rokas, L. N. A. Ries, T. Gabaldón, G. H. Goldman, N. P. Keller, C. Greco, Diversity of secondary metabolism in *Aspergillus nidulans* clinical isolates. *mSphere* **5**, e00156-20 (2020).

17. J. Yaegashi, M. B. Praseuth, S.-W. Tyan, J. F. Sanchez, R. Entwistle, Y.-M. Chiang, B. R. Oakley, C. C. C. Wang, Molecular genetic characterization of the biosynthesis cluster of a prenylated isoindolinone alkaloid aspernidine A in *Aspergillus nidulans*. *Org. Lett.* **15**, 2862–2865 (2013).

18. D. Zhang, S. M. Jennings, G. W. Robinson, C. D. Poulter, Yeast squalene synthase: Expression, purification, and characterization of soluble recombinant enzyme. *Arch. Biochem. Biophys.* **304**, 133–143 (1993).

19. H. Y. Chang, T. H. Cheng, A. H. Wang, Structure, catalysis, and inhibition mechanism of prenyltransferase. *IUBMB Life* **73**, 40–63 (2021).

20. M. A. Kennedy, M. Bard, Positive and negative regulation of squalene synthase (ERG9), an ergosterol biosynthetic gene, in Saccharomyces cerevisiae, in *Biochim. Biophys. Acta* **1517**, 177–189 (2001).

21. R. Do, R. S. Kiss, D. Gaudet, J. C. Engert, Squalene synthase: A critical enzyme in the cholesterol biosynthesis pathway. *Clin. Genet.* **75**, 19–29 (2009).

22. N. D. Lees, B. Skaggs, D. R. Kirsch, M. Bard, Cloning of the late genes in the ergosterol biosynthetic pathway of *Saccharomyces cerevisiae*—A review. *Lipids* **30**, 221–226 (1995).

23. C. Wen, Z. Zhang, Q. Shi, R. Niu, X. Duan, B. Shen, X. Li, Transcription factors *ZjMYB39* and *ZjMYB4* regulate farnesyl diphosphate synthase- and squalene synthase-mediated triterpenoid biosynthesis in jujube. *J. Agric. Food Chem.* **71**, 4599–4614 (2023).

24. A. Grover, G. Samuel, V. S. Bisaria, D. Sundar, Enhanced withanolide production by overexpression of squalene synthase in Withania somnifera. *J. Biosci. Bioeng.* **115**, 680–685 (2013).

25. R. Nagata, H. Suemune, M. Kobayashi, T. Shinada, K. Shin-Ya, M. Nishiyama, T. Hino, Y. Sato, T. Kuzuyama, S. Nagano, Structural basis for the prenylation reaction of carbazole-containing natural products catalyzed by squalene synthase-like enzymes. *Angew. Chem. Int. Ed. Engl.* **61**, e202117430 (2022).

26. K. Scherlach, J. Schuemann, H.-M. Dahse, C. Hertweck, Aspernidine A and B, prenylated isoindolinone alkaloids from the model fungus *Aspergillus nidulans*. *J. Antibiot. (Tokyo)* **63**, 375–377 (2010).

27. J.-F. Rontani, A. Mouzdahir, V. Michotey, P. Caumette, P. Bonin, Production of a polyunsaturated isoprenoid wax ester during aerobic metabolism of squalene by *Marinobacter squalenivorans* sp. nov. *Appl. Environ. Microbiol.* **69**, 4167–4176 (2003).

28. Y. Yamada, H. Motoi, S. Kinoshita, N. Takada, H. Okada, Oxidative degradation of squalene by Arthrobacter species. *Appl. Microbiol.* **29**, 400–404 (1975).

29. G. Wang, H. Ran, J. Fan, N. P. Keller, Z. Liu, F. Wu, W.-B. Yin, Fungal-fungal cocultivation leads to widespread secondary metabolite alteration requiring the partial loss-of-function VeA1 protein. *Sci. Adv.* **8**, eabo6094 (2022).

30. K. Dekermendjian, R. Shan, M. Nielsen, M. Stadler, O. Sterner, M. R. Witt, The affinity to the brain dopamine D_1 receptor in vitro of triprenyl phenols isolated from the fruit bodies of *Albatrellus ovinus*. *Eur. J. Med. Chem.* **32**, 351–356 (1997).

31. Q. Li, C. Chen, Y. He, M. Wei, L. Cheng, X. Kang, J. Wang, X. Hao, H. Zhu, Y. Zhang, Prenylated quinolinone alkaloids and prenylated isoindolinone alkaloids from the fungus *Aspergillus nidulans*. *Phytochemistry* **169**, 112177 (2020).

32. P. E. A. Teal, D. Jones, G. Jones, B. Torto, V. Nyasembe, C. Borgemeister, H. T. Alborn, F. Kaplan, D. Boucias, V. U. Lietze, Identification of methyl farnesoate from the hemolymph of insects. *J. Nat. Prod.* **77**, 402–405 (2014).

33. M. T. Nielsen, M. L. Klejnstrup, M. Rohlfs, D. C. Anyaogu, J. B. Nielsen, C. H. Gotfredsen, M. R. Andersen, B. G. Hansen, U. H. Mortensen, T. O. Larsen, *Aspergillus nidulans* synthesize insect juvenile hormones upon expression of a heterologous regulatory protein and in response to grazing by *Drosophila melanogaster* larvae. *PLOS ONE* **8**, e73369 (2013).

34. S. Mawa, I. Jantan, K. Husain, Isolation of terpenoids from the stem of Ficus aurantiaca griff and their effects on reactive oxygen species production and chemotactic activity of neutrophils. *Molecules* **21**, 9 (2016).

35. H. D. S. Siqueira, B. S. Neto, D. P. Sousa, B. S. Gomes, F. V. da Silva, F. V. M. Cunha, C. W. S. Wanderley, G. Pinheiro, A. G. F. Cândido, D. V. T. Wong, R. A. Ribeiro, R. C. P. Lima-Júnior, F. A. Oliveira, α-Phellandrene, a cyclic monoterpene, attenuates inflammatory response through neutrophil migration inhibition and mast cell degranulation. *Life Sci.* **160**, 27–33 (2016).

36. A. A. Brakhage, J. Schuemann, S. Bergmann, K. Scherlach, V. Schroeckh, C. Hertweck, Activation of fungal silent gene clusters: A new avenue to drug discovery. *Prog. Drug Res.* **66**, 3–12 (2008).

37. D. Wang, S. Jin, Q. Lu, Y. Chen, Advances and challenges in CRISPR/Cas-based fungal genome engineering for secondary metabolite production: A review. *J. Fungi* **9**, 362 (2023).

38. M. B. Quin, C. M. Flynn, C. Schmidt-Dannert, Traversing the fungal terpenome. *Nat. Prod. Rep.* **31**, 1449–1473 (2014).

39. R. A. González-Hernández, N. A. Valdez-Cruz, M. L. Macías-Rubalcava, M. A. Trujillo-Roldán, Overview of fungal terpene synthases and their regulation. *World J. Microbiol. Biotechnol.* **39**, 194 (2023).

40. R. Jaeger, E. Cuny, Terpenoids with special pharmacological significance: A review. *Nat. Prod. Commun.* **11**, 1373–1390 (2016).

41. H. Tao, L. Lauterbach, G. Bian, R. Chen, A. Hou, T. Mori, S. Cheng, B. Hu, L. Lu, X. Mu, M. Li, N. Adachi, M. Kawasaki, T. Moriya, T. Senda, X. Wang, Z. Deng, I. Abe, J. S. Dickschat, T. Liu, Discovery of non-squalene triterpenes. *Nature* **606**, 414–419 (2022).

42. K. Rose, A. Steinbüchel, Biodegradation of natural rubber and related compounds: Recent insights into a hardly understood catabolic capability of microorganisms. *Appl. Environ. Microbiol.* **71**, 2803–2812 (2005).

43. K. Bromann, M. Toivari, K. Viljanen, A. Vuoristo, L. Ruohonen, T. Nakari-Setälä, Identification and characterization of a novel diterpene gene cluster in *Aspergillus nidulans*. *PLOS ONE* **7**, e35450 (2012).

44. Y. Matsuda, T. Wakimoto, T. Mori, T. Awakawa, I. Abe, Complete biosynthetic pathway of anditomin: Nature's sophisticated synthetic route to a complex fungal meroterpenoid. *J. Am. Chem. Soc.* **136**, 15326–15336 (2014).

45. N. L. Brock, K. Huss, B. Tudzynski, J. S. Dickschat, Genetic dissection of sesquiterpene biosynthesis by *Fusarium fujikuroi*. *Chembiochem* **14**, 311–315 (2013).

46. D. Cox-Georgian, N. Ramadoss, C. Dona, C. Basu, Therapeutic and medicinal uses of terpenes. *Med. Plants*, 333–359 (2019).

47. M. L. Del Prado-Audelo, H. Cortés, I. H. Caballero-Florán, M. González-Torres, L. Escutia-Guadarrama, S. A. Bernal-Chávez, D. M. Giraldo-Gomez, J. J. Magaña, G. Leyva-Gómez, Therapeutic applications of terpenes on inflammatory diseases. *Front. Pharmacol.* **12**, 704197 (2021).

48. Q. Wang, J.-K. Liu, Q. Zhao, Q.-L. He, Mechanistic investigations of hirsutene biosynthesis catalyzed by a chimeric sesquiterpene synthase from *Steccherinum ochraceum*. *Fungal Genet. Biol.* **161**, 103700 (2022).

49. K. Shimizu, N. P. Keller, Genetic involvement of a cAMP-dependent protein kinase in a G protein signaling pathway regulating morphological and chemical transitions in *Aspergillus nidulans*. *Genetics* **157**, 591–600 (2001).

50. V. K. Okura, R. S. C. de Souza, S. F. de Siqueira Tada, P. Arruda, BAC-pool sequencing and assembly of 19 Mb of the complex sugarcane genome. *Front. Plant Sci.* **7**, 342 (2016).

51. S. Warming, N. Costantino, D. L. Court, N. A. Jenkins, N. G. Copeland, Simple and highly efficient BAC recombineering using galK selection. *Nucleic Acids Res.* **33**, e36 (2005).

52. M. T. Robey, R. Ye, J. W. Bok, K. D. Clevenger, M. N. Islam, C. Chen, R. Gupta, M. Swyers, E. Wu, P. Gao, P. M. Thomas, C. C. Wu, N. P. Keller, N. L. Kelleher, Identification of the first diketomorpholine biosynthetic pathway using FAC-MS technology. *ACS Chem. Biol.* **13**, 1142–1147 (2018).

53. K. D. Clevenger, R. Ye, J. W. Bok, P. M. Thomas, M. N. Islam, G. P. Miley, M. T. Robey, C. Chen, K. Yang, M. Swyers, E. Wu, P. Gao, C. C. Wu, N. P. Keller, N. L. Kelleher, Interrogation of benzomalvin biosynthesis using fungal artificial chromosomes with metabolomic scoring (FAC-MS): Discovery of a benzodiazepine synthase activity. *Biochemistry* **57**, 3237–3243 (2018).

54. P. J. Cavnar, K. Mogen, E. Berthier, D. J. Beebe, A. Huttenlocher, the actin regulatory protein HS1 interacts with Arp2/3 and mediates efficient neutrophil chemotaxis. *J. Biol. Chem.* **287**, 25466–25477 (2012).

55. K. P. Drees, J. M. Palmer, R. Sebra, J. M. Lorch, C. Chen, C. C. Wu, J. W. Bok, N. P. Keller, D. S. Blehert, C. A. Cuomo, D. L. Lindner, J. T. Foster, Use of multiple sequencing technologies to produce a high-quality genome of the fungus *Pseudogymnoascus destructans*, the causative agent of bat white-nose syndrome. *Genome Announc.* **4**, e00445-16 (2016).
